# Synthesis and Reactivity of Fluorinated Dithiocarboxylates to Prepare Thioamides—Effective Access to a 4-Styrenylthioamide-Cinchona Alkaloid Monomer

**DOI:** 10.3390/molecules28217333

**Published:** 2023-10-30

**Authors:** Aimar Gonzalo-Barquero, Bénédicte Lepoittevin, Jacques Rouden, Jérôme Baudoux

**Affiliations:** Laboratoire de Chimie Moléculaire et Thioorganique, UMR 6507, ENSICAEN, UNICAEN, CNRS, Normandie Université, 6 Bd du Maréchal Juin, 14050 Caen, France; aimar.gonzalo-barquero@ensicaen.fr (A.G.-B.); benedicte.lepoittevin@ensicaen.fr (B.L.); jacques.rouden@ensicaen.fr (J.R.)

**Keywords:** dithiocarboxylic acids, dithioesters, thioamides, grignard reagents, mitsunobu reaction

## Abstract

A simple and rapid access to fluorinated dithioesters was developed by a one-pot sequence corresponding to a Grignard reaction—Mitsunobu type substitution. These activated dithioesters have shown excellent reactivity in an aminolysis reaction from simple or more complex primary amines such as cinchona alkaloids. A stoichiometric amount of amine was sufficient to prepare various thioamides, including a 4-styrenylthioamide cinchonidine monomer, under environmentally friendly conditions, at room temperature, and in a very short time.

## 1. Introduction

**Figure 1:**
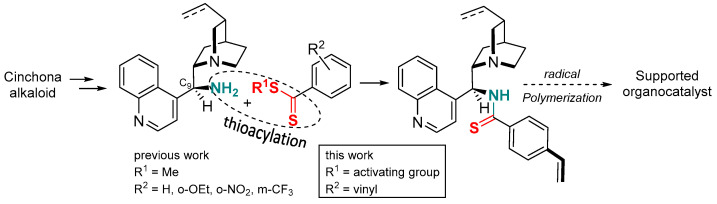
Synthesis and evaluation of dithioesters to access an organocatalyst based on a 4-styrenyl thioamide alkaloid monomer.

Dithiocarboxylic acids [1] or dithioesters [2] are common precursors of thioamides [3], a functional group that has found increasing uses over the last decade. In peptide chemistry, the thioamide function is a promising isostere of the amide group leading to interesting biological activities [4,5,6]. In organic chemistry, thioamide is particularly effective for the preparation of thiazoles or thiazolines and leads to higher yields compared to oxygen analogues [7,8,9]. In asymmetric synthesis, several groups including ours have described an improvement in the efficiency of thioamide organocatalysts in terms of yield and stereoselectivity compared to the amide analogues. For example, a thioamide-based catalyst has been successfully used in the addition of indoles to nitroalkenes [10], whereas proline-bearing thioamide functionality has led to a better result in various aldol reactions [11,12,13,14,15,16]. For the first time, we have used thioamide-substituted cinchona alkaloids for highly stereoselective decarboxylative Mannich or protonation reactions of malonic acid half-oxyesters affording α,β- and α-amino acid derivatives, respectively [17]. To prepare these organocatalysts, it should be noted that direct oxygen-sulfur exchange by the thionation of cinchona alkaloids bearing an amide function is ineffective and does not lead to the corresponding thioamides. For these specific scaffolds, the reaction of a methyldithioester with the 9-amino analogue of cinchona alkaloids led to thioamide-based organocatalysts in long reaction times (3–4 days) and moderate to good yields. In this context, we envisaged to synthesize (4-styrenyl)thioamide cinchona derivative which could be further polymerized to give a recyclable macromolecular catalyst [18]. Due to the sensitivity of the 4-styrenyl thioamide moiety and to ensure the larger-scale synthesis necessary for polymerization, we needed to optimize the synthesis of the dithioester precursor (Figure 1).

**Figure 2:**
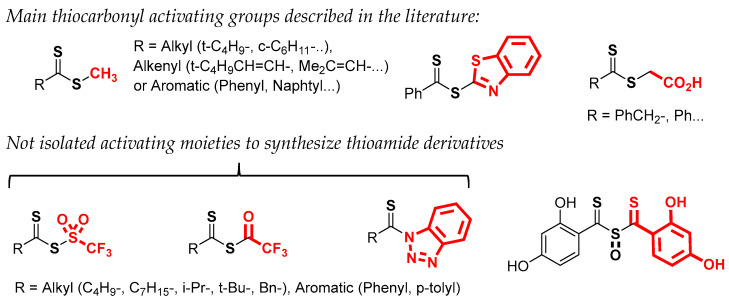
Activated dithioesters and derivatives.

Among various synthetic routes, the main method described to synthesize a dithioester uses a Grignard reagent which reacts with carbon disulfide to give a dithiocarboxylic acid as a key precursor [19,20,21]. After acid treatment, the recovery of the desired product will depend on the stability of the dithiocarboxylic acid. Very recently, Mitzel et al. reported the behavior of certain aryl compounds carrying this functionality [22]. These reactive species often generate different types of oligomers or oxidative decomposition products. In the case of aryl dithiocarboxylic acids, the ortho substitution facilitates its isolation in pure form due to the steric hindrance which limits the formation of oligomers. Lampkins et al. synthesized various 4-alkoxy-dithionaphthoic acids in good yield and confirmed their stability [23]. The same authors used these dithiocarboxylic acids to prepare methyl dithioesters as precursors of thioamides. Nevertheless, the aminolysis with primary or secondary amines in the presence of DMAP was not possible for these compounds. To improve the reactivity of such aliphatic dithioesters (methyl or ethyl), various activating moieties have been tested (Figure 2) [24,25,26,27,28], mention should be made of benzothiazolyl [24], sulfinyl bisthiobenzoyl [25,26], or thioglycolic acid [7,8,27]. Among these, S-thiobenzoylglycolic acid represents an interesting choice. The corresponding dithioester can react with a primary amine in the presence of pyridine and triethylamine to generate the desired thioamide.

**Scheme 1:**
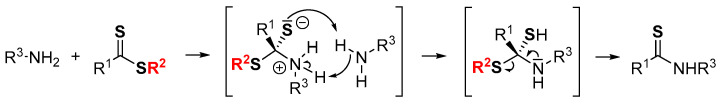
Proposed mechanism for the formation of thioamides from dithioesters and amines.

A strong point of these activating reagents is their electron-withdrawing character. Indeed, the proposed mechanism involves first the reversible nucleophilic addition of the primary amine to give a zwitterionic intermediate (Scheme 1). Depending on the nature of the R^2^ group, this zwitterionic intermediate is able to block or accelerate the thioamide formation process. The addition of a tertiary amine has no real influence on the reaction rate, unlike the amount of primary amine. Thus, an excess of primary amine is required to protonate the thiolate and deprotonate the ammonium moiety simultaneously, according to Scheme 1 [29,30,31].

Therefore, according to this mechanism, amine hindrance plays a crucial role in thioamide formation. Thus, as part of our research in organocatalysis, this work aims to study the reactivity of newly activated aryldithioesters and styrenyldithioesters with more complex chiral primary amines such as cinchona alkaloids. In these cases, their original structure and the presence of a basic site (quinuclidine) accentuate the difficulty of developing efficient and rapid access to functionalized and supported organocatalysts.

## 2. Results

### 2.1. Synthesis of Dithioesters and Thionoesters

**Scheme 2:**
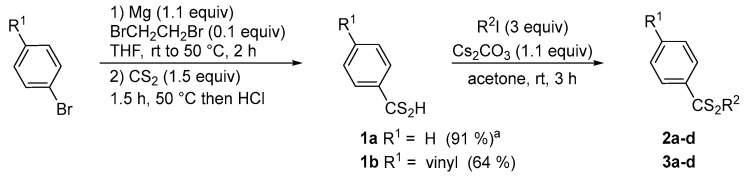
Synthesis of dithioesters **2a**–**d** and **2b**–**d** by Grignard reaction. ^a^ From PhMgBr.

Firstly, the synthesis of dithioesters was studied using two different strategies. Under standard conditions, Grignard reagents were directly used from commercially available (R = H) or prepared from an aryl bromide and generated in situ (R = vinyl). Subsequently, the organomagnesium compound reacted with carbon disulfide (1.5 equiv) to form magnesium dithiocarboxylate. After acid treatment, the corresponding dithiocarboxylic acids **1a** and **1b** were isolated in 91% and 64% yield, respectively (Scheme 2). By the presence of a suitable base, these dithioacids reacted with an excess of iodomethane to generate the dithioesters **2a**–**3a**. Several bases were tested such as K_2_CO_3_, Na_2_CO_3_, Cs_2_CO_3,_ and Et_3_N. After optimization, the best yield was obtained using a stoichiometric quantity of cesium carbonate and iodomethane (3 equiv), at room temperature in acetone (or butanone). Under similar conditions, different alkylating agents (R^2^I) were used with dithioacids **1a**–**b**, which made it possible to synthesize several dithioesters **2b**–**2d** and **3b**–**3d** to compare the effectiveness of this sequence (Table 1).

**Scheme 3:**
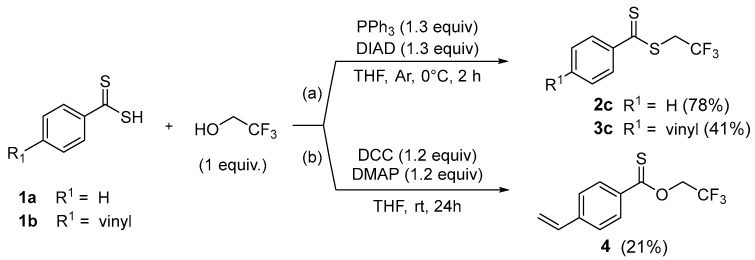
Preparation of dithioesters **2c**–**3c** or thionoester **4** by standard Mitsunobu or Steglich reaction from dithiocarboxylic acids **1a**–**1b**.

From dithiocarboxylic acid **1a**, yields close to 70–80% were obtained with aliphatic (methyl or ethyl) and perfluorinated agents. Comparatively, the presence of a vinyl unit, which is more difficult to handle, causes a logical decrease in yield after acid treatment. After purification, the dithioesters **3a**–**d** were isolated in moderate yields from 21% to 53%. Consequently to the low yield obtained for dithioester **3c**, we evaluated the standard Mitsunobu reaction conditions to alkylate dithiocarboxylic acids **1a**–**b** with trifluoroethanol (Scheme 3(a)) [32].

Thus, we used a stoichiometric amount of a dithiocarboxylic acid and 2,2,2-trifluoroethanol in the presence of a slight excess of PPh_3_ and DIAD in THF at 0 °C. After 2 h, we observed a good yield of 78% in dithioester **2c** which is slightly higher than the alkylation reaction (72%). With the more sensitive vinylic unit, a significant proportion of dithioester **3c** was isolated to reach 41% yield (instead of 21% for the alkylation reaction) confirming the potential of this procedure. In parallel, we prepared thionoester **4** as a potential precursor of thioamides using DCC (1.2 equiv) and DMAP (1.2 equiv) in THF at room temperature (Scheme 3(b)). After 24 h, dithiocarboxylic acid **1b** afforded desired compound **4** in 21% yield.

Dithiocarboxylic acids are particularly delicate to store and handle with the rapid appearance of by-products consistent with the literature [22]. Thus, we evaluated a one-pot reaction starting from 4-vinylbenzene bromide, an easy-to-store commercial reagent. The use of an organometallic reagent as a nucleophile in a Mitsunobu-type substitution is a poorly described method. A few examples have described the use of a zinc [33,34] or lithium [35] salt but the reactivity of an organomagnesium reagent remains unknown (Table 2).

In this sequence, we exploited the previously optimized reaction to generate the Grignard reagent which was engaged directly with the 2,2,2-trifluoroethanol without acid treatment. With two equivalents of PPh_3_ and DIAD, the magnesium salts (R = H or vinyl) gave dithioesters **2c** and **3c** in 71% and 40%, respectively. Ethyl and benzyl alcohols were tested in this sequence with similar efficiency affording the corresponding dithioester **2b** and **2e** in good yield. Compared to isolated dithiocarboxylic acids which required acid treatment and tedious extraction, inefficient purification to be avoided by column chromatography, and two working days to generate the corresponding dithioester, we observed similar yields in less than one day for these two steps of the one-pot reaction. Having in hand an efficient and easy procedure to access dithioesters **2a**–**d** and **3a**–**d** and thionoester **4**, we started the aminolysis of these model substrates.

### 2.2. Aminolysis of Dithioesters and Thionoesters

**Figure 3:**
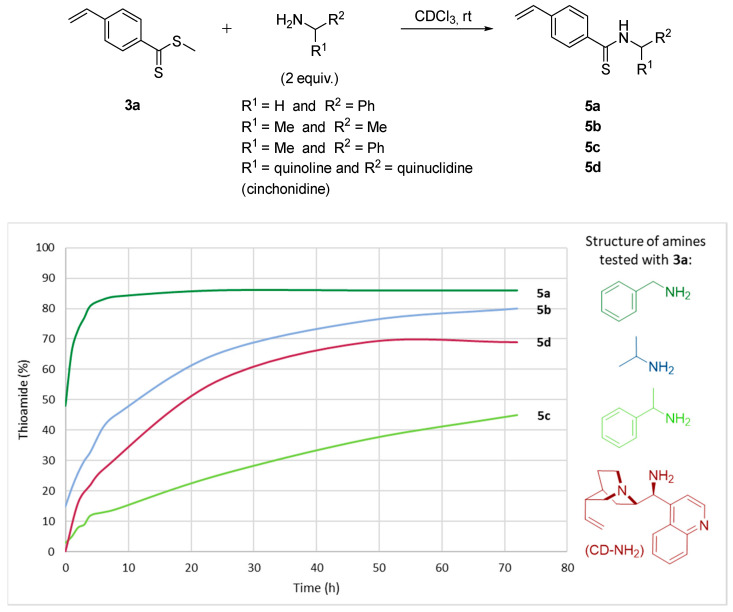
^1^H NMR monitoring reaction progress between a primary amine on the dithioester **3a** in chloroform-d.

As a reference reaction, the reactivity of the methylated dithioester **3a** was first evaluated through various nucleophiles carrying a primary amine, the ^1^H NMR monitoring is presented in Figure 3. First, benzylamine was compared to α-branched primary amines such as 2-propylamine and two chiral amines, α-methylbenzylamine and 9-aminocinchonidine. In the presence of an excess of benzylamine (2 equiv), a very rapid conversion to thioamide **5a** was observed, reaching 80% after 4 h of reaction. On the contrary, isopropylamine reacts more slowly to give 60% of thioamide **5b** after 20 h and 80% after 72 h of reaction. The use of chiral α-methylbenzylamine confirmed this trend with a clear loss of reactivity to generate only 45% of thioamide **5c** after 72 h. Thus, Figure 3 reveals poor reactivity of amines having an alpha substitution of nitrogen. Nevertheless, the primary amine derivative of cinchonidine showed very similar kinetics to isopropylamine with 50% of thioamide **5d** after 20 h to reach a maximum of 70% after 50 h. This difference may be related to the presence of the quinuclidine moiety carrying a basic tertiary amine nearby.

The reaction conditions were then optimized using one equivalent of α-methylbenzylamine up to 4 equivalents relative to the dithioester **3a**. With a stoichiometric ratio, the reactivity decreases significantly compared to the same reaction using two equivalents of amine. After 50 h, a low conversion of 30% was observed which is consistent with the mechanism proposed in Scheme 1. Beyond the use of two equivalents, the reaction rate increases only slightly, the addition of a large excess of amine does not represent any real synthetic interest. Finally, the influence of concentration and temperature was also studied. Thus, the most effective concentration of dithioester **3a** was established at 0.45 mol/L in a concentration range between 0.9 mol/L and 0.09 mol/L, while non-isolated by-products were observed when the temperature was increased to 60 °C.

**Figure 4:**
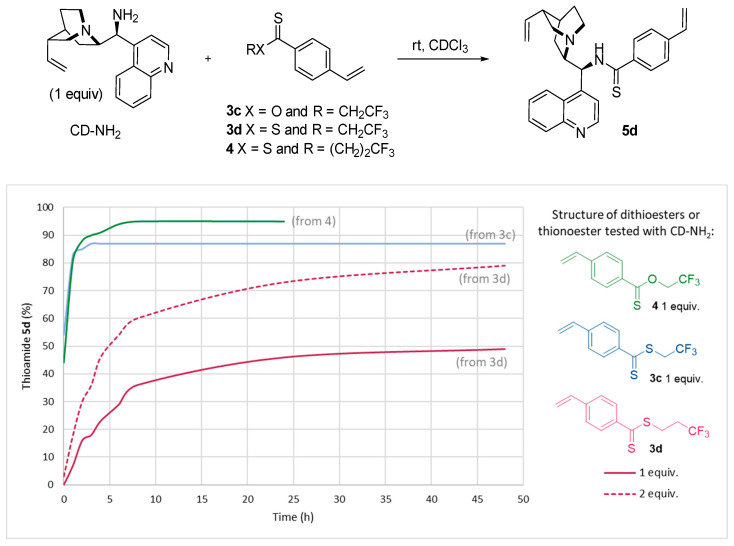
^1^H NMR monitoring reaction progress between CD-NH_2_ and dithioesters **3c**–**d** or **4**.

Under optimized conditions, dithioesters **3c**–**d** and thionoester **4** were reacted with the amine derivative of cinchonidine (Figure 4). Here, the goal was to establish the most effective (and easily accessible) reagent. In this context, we also wanted to develop a simple method consuming a smaller quantity of nucleophiles. To this end, we evaluated the use of one equivalent of cinchonidine at room temperature.

Compared to a simple methyl, the electronic effects caused by a terminal CF_3_ unit is clearly positive in the addition of cinchonidine (Figure 4). A stoichiometric amount of the dithioester **3c** led to an excellent yield of 83% after only one hour of reaction at room temperature, probably due to the higher electrophilicity of the thiocarbonyl and the proximity of electronegative fluorine atoms. A significant difference was observed with dithioester **3d** which presents a longer flexible chain between the thiocarbonyl group and the fluorine atoms to lead to only 7% conversion after 1 h. Under these conditions, a maximum of 50% was detected by ^1^H NMR after 2 days of reaction. To confirm this result, excess of the dithioester **3d** (2 equiv) was added to the cinchondine-NH_2_ to provide 50% of thioamide after 5 h at room temperature. After 2 days, a conversion of 80% was observed. Interestingly, thionoester **4** incorporating a trifluoroethyl group showed a very similar reactivity to the dithioester **3c**, a very rapid substitution of 83% was calculated after 1 h of reaction at room temperature reaching 95% after 8 h.

## 3. Discussion

**Scheme 4:**
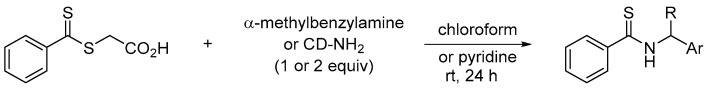
Reactivity of S-thiobenzoylglycolic acid towards a chiral primary amine.

To establish the potential of the dithioester **3c** in comparison to previous studies, we evaluated a commercial dithioester regularly cited in the literature for the synthesis of thioamides, namely S-thiobenzoylglycolic acid [27]. This reagent was reacted with α-methylbenzylamine or 9-aminocinchonidine in chloroform or pyridine (Scheme 4).

In pyridine, a low conversion of 49% was calculated for α-methylbenzylamine after 5 h at room temperature to reach 72% after 24 h. In chloroform, the addition of this amine caused a precipitate very quickly. In this case, the addition of 2 equivalents of α-methylbenzylamine was necessary to observe a conversion of 46% after 4 h and 85% after 20 h (the concentration of the medium is very important to obtain a good conversion). From the same stoichiometry, thioamide **5c** was isolated with a yield of 84% after 3 days of reaction with dithioester **3a** while a yield of 78% and 69% was observed for **5a** and **5b**, respectively (see experimental section).

**Scheme 5:**
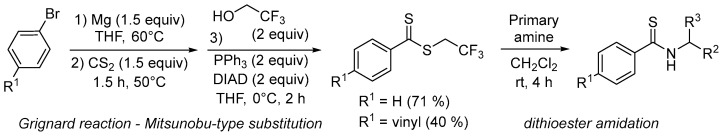
One-pot sequence optimized in this study.

In contrast, cinchonidine-NH_2_ (1 equiv) did not react with S-thiobenzoylglycolic acid in chloroform or pyridine at room temperature. A very low conversion of 4% to thioamide was observed after 24 h of reaction. The low reactivity of this chiral primary amine with S-thiobenzoylglycolic acid confirmed the potential of the trifluorinated dithioester **3c** evaluated in this study. Indeed, from a one-pot sequence, halogenated aromatic compounds provided fluorinated dithioesters in a few hours and their reactions with a (chiral) primary amine efficiently led to a thioamide (Scheme 5). Thus, the reaction sequence developed herein is well-suited particularly when direct thionation methods of amides failed.

Fluorinated dithioesters showed excellent reactivity in an aminolysis reaction with a stoichiometric amount of 9-amino-9-deoxy-cinchonidine. Precisely, two equivalents of methylated dithioester **3a** are necessary to prepare thioamide **5d** with a maximum yield of 70% after 48 h while one equivalent of fluorinated dithioester **3c** provides 90% in approximately 2 h. From these results, the overall sequence was used to synthesize 4-styrenylthioamide cinchonidine as a reactive monomer necessary for our ongoing project on polymer-supported organocatalysis.

## 4. Materials and Methods

### 4.1. General Information

All reagents were purchased from Acros Organics, Sigma Aldrich, Alfa Aesar, TCI, or Fluka and were used without further purification and used as received. Solvents were used in RPE grade without further purification. Anhydrous solvents were obtained from a PURESOLV SPS400 apparatus developed by Innovative Technology Inc. (Oldham, UK). ^1^H and ^13^C NMR spectra were recorded on a Bruker Avance III 500 MHz. Samples were dissolved in an appropriate deuterated solvent (CDCl_3_). The chemical shifts (δ) are expressed in ppm and coupling constants are indicated in Hz. Abbreviations for signal coupling are as follows: s = singlet; d = doublet; t = triplet; q = quartet; m = multiplet; br = broad signal). To assign the signals to the different proton and carbon atoms, additional 2D NMR experiments (COSY, HSQC, HMBC) and NOESY experiments were performed. 1,3,5-trimethoxybenzene was used as an internal standard to determine ^1^H NMR yield. High-resolution mass spectra (HRMS) were performed on Acquity UPLC HClass Xevo G2-XS QTof (WATERS) by electrospray ionization (ESI). Infrared (IR) spectra were recorded with a Perkin Elmer 16 PC FTIR ATR spectrometer, using the pure product (oil or solid). Thin Layer Chromatography (TLC) was run on pre-coated aluminum plates of silica gel 60 F-254 (Merck). Flash chromatography was performed on a silica gel column (Merck silica gel, 40–63 µm) using air pressure.

### 4.2. Experimental

#### 4.2.1. General Procedure for the Preparation of Dithioesters **2a**–**d** by Alkylation of Dithiocarboxylic Acid **1a**

To an oven-dried flask purged with argon was added a solution of phenylmagnesium bromide 1M in THF (8.27 mL, 8.27 mmol) and diluted with dry THF (80 mL). Carbon disulfide (0.75 mL, 12.41 mmol) was slowly added at room temperature and the reaction mixture was stirred at 50 °C for 1.5 h. The resulting mixture was allowed to cool down to room temperature and 10 mL of water was added to quench the reaction. The mixture was evaporated under reduced pressure to remove the THF. The aqueous solution was diluted with water (50 mL) and washed with DCM twice, then acidified with 1M HCl. Once acidified, a pink-colored precipitate appeared. The mixture was extracted three times with DCM and the solvent was evaporated under reduced pressure. Freshly prepared dithiocarboxylic acid (1.16 g, 7.52 mmol) was dissolved in acetone (26 mL). CsCO_3_ (2.70 g, 8.27 mmol), the corresponding alkyl halide (3 equiv.) was then added and the mixture was stirred at room temperature overnight. The resulting dithioester was purified by column chromatography using pentane as solvent.

#### 4.2.2. Characterization of Dithioesters

*Methyl dithiobenzoate* (**2a**) (CAS: 2168-78-7). Dithioester **2a** was obtained as a red liquid. Yield: 62%. ^1^H NMR (500 MHz, CDCl_3_): δ = 8.03–8.02 (m, 2H), 7.54 (tt, *J* = 7.4, 1.2 Hz, 1H), 7.42–7.38 (m, 2H), 2.79 (3H, s). ^13^C NMR (125 MHz, CDCl_3_): δ = 228.6, 144.7, 132.1, 128.1, 126.6, 20.5. HMRS (ASAP^+^) C_8_H_9_S_2_ [(M+H)^+^]: calculated: 169.0146, found: 169.0145. IR: 1229, 686 cm^−1^.

*Ethyl dithiobenzoate* (**2b**) (CAS: 936-63-0). Dithioester **2b** was obtained as a red liquid. Yield: 72%. ^1^H NMR (500 MHz, CDCl_3_): δ = 8.01–7.99 (m, 2H), 7.51 (tt, *J* = 7.4, 1.2 Hz, 1H), 7.40–7.37 (m, 2H), 3.37 (q, *J* = 7.6 Hz, 2H), 1.42 (t, *J* = 7.6 Hz, 3H). ^13^C NMR (125 MHz, CDCl_3_): δ = 228.7, 145.3, 132.3, 128.4, 126.9, 31.6, 12.4. HMRS (ASAP^+^) C_9_H_11_S_2_ [(M+H)^+^]: calculated: 183.0302, found: 183.0294. IR: 1205, 688 cm^−1^.

*2,2,2-trifluoroethyl dithiobenzoate* (**2c**). Dithioester **2c** was obtained as a red liquid. Yield: 72%. ^1^H NMR (500 MHz, CDCl_3_): δ = 8.04–8.02 (m, 2H), 7.59 (tt, *J* = 7.4, 1.2 Hz, 1H), 7.45–7.41 (m, 2H), 4.26 (q, *J* = 9.9 Hz, 2H). ^13^C NMR (125 MHz, CDCl_3_): δ = 223.6, 144.3, 133.2, 128.6, 127.3, 124.8 (q, *J* = 276.8 Hz), 38.0 (q, *J* = 38.0 Hz). ^19^F NMR (471 MHz, CDCl_3_): δ = −64.42 (t, *J* = 9.9 Hz). HMRS (ASAP^+^) C_9_H_8_F_3_S_2_ [(M+H)^+^]: calculated: 237.0020, found: 237.0019. IR: 1305, 1269, 1235, 1210, 1129, 1048, 646 cm^−1^.

*2,2,2-trifluoropropyl dithiobenzoate* (**2d**). Dithioester **2d** was obtained as a red liquid. Yield: 78%. ^1^H NMR (500 MHz, CDCl_3_): δ = 8.04–8.02 (m, 2H), 7.57 (tt, *J* = 7.3, 1.3 Hz, 1H), 7.43–7.40 (m, 2H), 3.60–3.57 (m, 2H), 2.62–2.53 (m, 2H). ^13^C NMR (125 MHz, CDCl_3_): δ = 226.8, 144.75, 132.9, 128.6, 127.0, 126.1 (q, *J* = 277.3 Hz), 32.5 (q, *J* = 29.3 Hz), 28.7 (q, *J* = 3.2 Hz). ^19^F NMR (471 MHz, CDCl_3_): δ = −66.15 (t, *J* = 9.9 Hz). HMRS (ASAP^+^) C_10_H_10_F_3_S_2_ [(M+H)^+^]: calculated: 251.0176, found: 251.0176. IR: 1241, 1206, 1092, 687 cm^−1^.

*Benzyl dithiobenzoate* (**2e**). Dithioester **2e** was obtained as a red liquid. Yield: 59%. ^1^H NMR (600 MHz, CDCl_3_): δ = 8.02 (d, *J* = 8.4; 1.1 Hz, 2H), 7.56 (tt, *J* = 7.4, 1.1 Hz, 1H), 7.41–7.38 (m, 4H), 7.37–7.34 (m, 2H), 7.31 (tt, *J* = 7.2, 1.1 Hz, 1H), 4.65 (s, 2H). ^13^C NMR (150 MHz, CDCl_3_): δ = 227.8, 144.8, 135.1, 132.5, 129.4, 128.8, 128.5, 127.9, 127.0, 42.4. HMRS (ASAP^+^) C_14_H_13_S_2_ [(M+H)^+^]: calculated: 245.0459, found: 245.0451. IR: 1225, 685 cm^−1^.

#### 4.2.3. General Procedure for the Preparation of Dithioesters **3a**, **b**, **d** by Alkylation of Dithiocarboxylic Acid **1b**

To an oven-dried flask purged with argon, magnesium turnings were added (291 mg, 12 mmol) with 2 mL of dry THF. Then, 1,2-dibromoethane (205 mg, 1.09 mmol) was added. A solution of 4-bromostyrene (2 g, 10.9 mmol) in dry THF (5 mL) was added dropwise at room temperature. The reaction mixture was then diluted with 16 mL of dry THF and stirred at 60 °C for 2 h. The reaction mixture was allowed to cool to room temperature and carbon disulfide (0.98 mL, 16.35 mmol) was slowly added. The reaction mixture was stirred at 50 °C for 1.5 h. The resulting mixture was allowed to cool to room temperature and 10 mL of water was added to quench the reaction. The mixture was evaporated under reduced pressure to remove the THF. The aqueous solution was diluted with water (50 mL) and washed with DCM twice, then acidified with 1M HCl. After the acidification of the mixture, a pink-colored precipitate appeared. The mixture was extracted three times with DCM and the solvent was evaporated under reduced pressure. Freshly prepared dithiocarboxylic acid (1.26 g, 6.98 mmol) was dissolved in acetone (24 mL). CsCO_3_ (2.50 g, 7.68 mmol), hydroquinone (77.08 mg, 0.70 mmol), and the corresponding alkyl halide (3 equiv.) were then added and the mixture was stirred at room temperature overnight. The resulting dithioester was purified by column chromatography using pentane as solvent.

#### 4.2.4. Characterization of 4-Vinyldithioesters

*Methyl 4-vinyldithiobenzoate* (**3a**). Dithioester **3a** was obtained as a red solid. Yield: 35%. Mp: 25 °C. ^1^H NMR (500 MHz, CDCl_3_): δ = 8.04 (d, *J* = 8.5 Hz, 2H), 7.41 (d, *J* = 8.0 Hz, 2H), 6.74 (dd, *J* = 17.6, 11.0 Hz, 1H), 5.88 (d, *J* = 17.6 Hz, 2H), 5.40 (d, *J* = 11.0 Hz, 2H), 2.78 (s, 3H). ^13^C NMR (125 MHz, CDCl_3_): δ = 228.1, 144.1, 141.6, 136.0, 127.3, 126.2, 116.4, 20.7. HMRS (ASAP^+^) C_10_H_11_S_2_ [(M+H)^+^]: calculated: 195.0302, found: 195.0299. IR: 1237, 637cm^−1^.

*Ethyl 4-vinyldithiobenzoate* (**3b**). Dithioester **3b** was obtained as a red liquid. Yield: 49%. ^1^H NMR (500 MHz, CDCl_3_): δ = 8.01 (d, *J* = 8.4 Hz, 2H), 7.40 (d, *J* = 8.4 Hz, 2H), 6.73 (dd, *J* = 17.6, 10.9 Hz, 1H), 5.87 (d, *J* = 17.6 Hz, 1H), 5.39 (d, *J* = 10.9 Hz, 1H), 3.37 (q, *J* = 7.5 Hz, 2H), 1.42 (t, *J* = 7.5 Hz, 3H). ^13^C NMR (125 MHz, CDCl_3_): δ = 227.3, 144.3, 141.6, 136.1, 127.3, 126.1, 116.3, 31.5, 12.4. HMRS (ASAP^+^) C_11_H_13_S_2_ [(M+H)^+^]: calculated: 209.0459, found: 209.0458. IR: 2953, 2922, 2852, 1212 cm^−1^.

*2,2,2-trifluoropropyl 4-vinyldithiobenzoate* (**3d**). Dithioester **3d** was obtained as a red solid. Yield: 53%. Mp: 50 °C. ^1^H NMR (500 MHz, CDCl_3_): δ = 8.01 (d, *J* = 8.4 Hz, 2H), 7.42 (d, *J* = 8.4 Hz, 2H), 6.74 (dd, *J* = 17.6, 10.9 Hz, 1H), 5.88 (d, *J* = 17.6 Hz, 1H), 5.41 (d, *J* = 10.9 Hz, 1H), 3.59–3.56 (dt, *J* = 7.6, 1.2 Hz, 2H), 2.65–2.52 (m, 2H). ^13^C NMR (125 MHz, CDCl_3_): δ = 225.4, 143.7, 142.1, 135.9, 127.4, 126.3, 126.1 (q, *J* = 277.4 Hz) 116.8, 32.5 (q, *J* = 28.9 Hz) 28.5 (q, *J* = 3.2 Hz). ^19^F NMR (471 MHz, CDCl_3_): δ = −66.15 (t, *J* = 10.4 Hz). HMRS (ASAP^+^) C_12_H_12_F_3_S_2_ [(M+H)^+^]: calculated: 277.0333, found: 277.0339. IR: 2921, 1235, 1217, 1134, 639 cm^−1^.

#### 4.2.5. Synthesis of 2,2,2-Trifluoroethyl 4-Vinyldithiobenzoate **3c** by Cascade Reaction

Grignard reaction: Magnesium turnings (0.44 g, 18.30 mmol) were added to an oven-dried flask. The flask was dried at 200 °C (with a heat gun) and purged with argon. Next, 2 mL of dry THF and 1,2-dibromoethane (0.14 mL, 1.64 mmol) were added and the mixture was vigorously stirred. A solution of 4-bromostyrene (2.14 mL, 16.39 mmol) in dry THF (6 mL) was prepared in an oven-dried flask and 2 mL of this solution was added to the magnesium at room temperature. The rest of the solution was added dropwise after the beginning of the reaction (gentle bubbling). The reaction mixture was then diluted with 24 mL of dry THF and stirred at 60 °C for 2 h. The reaction mixture was allowed to cool to room temperature and carbon disulfide (1.48 mL, 24.59 mmol) was added dropwise. The reaction mixture was stirred at 50 °C for 1.5 h.

Mitsunobu reaction: A solution of PPh_3_ (8.60 g, 32.78 mmol), DIAD (6.44 mL, 32.78 mmol), 2,2,2-trifluoroethanol (2.48 mL, 32.78 mmol), and hydroquinone (0.18 g, 1.64 mmol) was prepared in 35 mL of dry THF at 0 °C. The solution was stirred for 30 min at 0 °C and the crude mixture containing the Grignard product was added. The solution was stirred for 2 h at 0 °C and the product was purified by column chromatography using pentane as solvent. Dithioester **3c** was obtained as a red solid. Yield: 2.02 g (47%). Mp: 40 °C.

^1^H NMR (500 MHz, CDCl_3_): δ = 8.03 (d, *J* = 8.4 Hz, 2H), 7.44 (d, *J* = 8.4 Hz, 2H), 6.75 (dd, *J* = 17.6, 10.9 Hz, 1H), 5.90 (d, *J* = 17.6 Hz, 1H), 5.39 (d, *J* = 10.9 Hz, 1H), 4.25 (q, *J* = 9.9 Hz, 2H). ^13^C NMR (125 MHz, CDCl_3_): δ = 222.2, 143.3, 142.6, 135.9, 127.8, 126.4, 124.9 (q, *J* = 276.7 Hz), 117.1, 38.0 (q, *J* = 32.9 Hz). ^19^F NMR (471 MHz, CDCl_3_): δ = −64.41 (t, *J* = 9.9 Hz). HMRS (ASAP^+^) C_11_H_10_F_3_S_2_ [(M+H)^+^]: calculated: 263.0176, found: 263.0184. IR: 2911, 1302, 1231, 1054, 638 cm^−1^.

#### 4.2.6. Synthesis of 2,2,2-Trifluoropropyl 4-Vinylthiobenzoate **4**

Freshly prepared dithiocarboxylic acid **1b** (500 mg, 2.77 mmol), trifluoroethanol (0.42 mL, 5.55 mmol), DMAP (407 mg, 3.33 mmol), and DCC (687 mg, 3.33 mmol), were dissolved in THF (8.21 mL) and the mixture was stirred at room temperature for 24 h. The product was purified by flash chromatography (pentane). Thionoester **4** was obtained as a yellow liquid. Yield: 125 mg (18%).

^1^H NMR (500 MHz, CDCl_3_): δ = 8.17 (d, *J* = 8.6 Hz, 2H), 7.43 (d, *J* = 8.6 Hz, 2H), 6.76 (dd, *J* = 17.6, 10.9 Hz, 1H), 5.90 (d, *J* = 17.6 Hz, 1H), 5.43 (d, *J* = 10.9 Hz, 1H), 4.99 (q, *J* = 8.3 Hz, 2H). ^13^C NMR (125 MHz, CDCl_3_): δ = 208.3, 142.4, 136.3, 135.9, 129.5, 126.1, 123.2 (q, *J* = 275.5 Hz), 117.1, 67.1 (q, *J* = 36.4 Hz). ^19^F NMR (471 MHz, CDCl_3_): δ = −72.79 (t, *J* = 8.3 Hz). HMRS (ASAP^+^) C_11_H_10_OF_3_S [(M+H)^+^]: calculated: 247.0404, found: 247.0401. IR: 2953, 1276, 1199, 1164 cm^−1^.

#### 4.2.7. General Procedure for Thioamide **5a**–**c** Synthesis

Dithioester **3a** (176 mg, 0.91 mmol,) was dissolved in DCM (2 mL) with the corresponding amine (1.81 mmol) and the mixture was stirred at room temperature for three days. The thioamide obtained was purified by column chromatography (cyclohexane:AcOEt, 90:10).

*N-benzyl-4-vinylbenzothioamide* (**5a**). Thioamide **5a** was obtained as a yellow solid. Yield: 78%. Mp: 81 °C. ^1^H NMR (500 MHz, CDCl_3_): δ = 7.83–7.76 (br, 1H), 7.73 (d, *J* = 8.4 Hz, 2H), 7.41–7.33 (m, 7H), 6.71 (dd, *J* = 17.6, 10.9 Hz, 1H), 5.82 (d, *J* = 17.6 Hz, 1H), 5.35 (d, *J* = 10.9 Hz, 1H), 4.98 (d, *J* = 5.2 Hz, 2H). ^13^C NMR (125 MHz, CDCl_3_): δ = 198.4, 140.5, 136.3, 135.9, 129.1, 128.4, 128.3, 127.1, 126.3, 116.0, 51.0. HMRS (ASAP^+^) C_16_H_16_NS [(M+H)^+^]: calculated: 254.1003, found: 254.1002. IR: 3308, 1521, 1500, 1326 cm^−1^.

*4-vinyl-N-propan-2-ylbenzothioamide* (**5b**). Thioamide **5b** was obtained as a yellow solid. Yield: 69%. Mp: 76 °C. ^1^H NMR (500 MHz, CDCl_3_): δ = 7.63 (d, *J* = 8.5 Hz, 2H), 7.70–7.49 (br, 1H), 7.33 (d, *J* = 8.6 Hz, 2H), 6.68 (dd, *J* = 17.6, 10.9 Hz, 1H), 5.78 (d, *J* = 17.6 Hz, 1H), 5.32 (d, *J* = 10.9 Hz, 1H), 4.76 (sextet, *J* = 6.6 Hz, 1H), 1.33 (d, *J* = 6.7 Hz, 6H). ^13^C NMR (125 MHz, CDCl_3_): δ = 197.2, 141.2, 140.2, 135.9, 127.0, 126.2, 115.9, 48.1, 21.6. HMRS (ASAP^+^) C_12_H_16_NS [(M+H)^+^]: calculated: 206.1003, found: 206.1006. IR: 3191, 1521, 1502, 1363 cm^−1^.

*4-vinyl-N-(1-phenylethyl)benzothioamide* (**5c**). Thioamide **5c** was obtained as a yellow oil. Yield: 84%. Mp 76 ˚C. ^1^H NMR (500 MHz, CDCl_3_): δ = 7.78–7.72 (br, 1H), 7.70 (d, *J* = 8.7 Hz, 2H), 7.43–7.37 (m, 6H), 7.33 (tt, *J* = 7.1, 1.5 Hz, 1H), 6.71 (dd, *J* = 17.6, 10.9 Hz, 1H), 5.92 (quintuplet, *J* = 7.2 Hz, 1H), 5.82 (d, *J* = 17.6 Hz, 1H), 5.35 (d, *J* = 10.9 Hz, 1H), 1.71 (d, *J* = 7.0 Hz, 3H). ^13^C NMR (125 MHz, CDCl_3_): δ = 197.4, 141.5, 141.0, 140.4, 135.9, 129.0, 128.4, 128.0, 127.1, 126.7, 126.3, 116.0, 55.2, 20.3. HMRS (ASAP^+^) C_17_H_18_NS [(M+H)^+^]: calculated: 268.1160, found: 268.1162. IR: 3235, 3028, 2974, 1516, 1493, 1367 cm^−1^.

#### 4.2.8. Synthesis of *N*-(9-Deoxyepicinchonidin-9-yl)-4-vinylbenzothioamide **5d**

Dithioester **3c** (200 mg, 0.76 mmol) was dissolved in DCM (1.7 mL) and 9-amino-(9-deoxy)epi-cinchonidine (224 mg, 0.76 mmol) was added. The mixture was stirred at room temperature for four hours. The crude was purified by column chromatography (DCM:MeOH, 95:5) to yield thioamide **5d** as a yellow solid. Yield: 313 mg (93%). mp: 92 ˚C. ^1^H NMR (500 MHz, CDCl_3_): δ = 9.42–9.01 (br, 1H), 8.87 (d, *J* = 4.6 Hz, 1H), 8.45 (d, *J* = 8.2 Hz, 1H), 8.15 (dd, *J* = 8.5, 1.0 Hz, 1H), 7.79 (d, *J* = 8.4 Hz, 2H), 7.73 (ddd, *J* = 8.2, 7.0, 1.2 Hz, 1H), 7.64 (t, *J* = 7.3 Hz, 1H), 7.45 (d, *J* = 4.5 Hz, 1H), 7.42 (d, *J* = 8.4 Hz, 2H), 6.72 (dd, *J* = 17.6, 10.9 Hz, 1H), 5.82 (d, *J* = 17.6 Hz, 1H), 5.79–5.71 (br, 1H), 5.67 (ddd, *J* = 17.3, 10.2, 7.3 Hz, 1H), 5.34 (d, *J* = 11.0 Hz, 1H), 4.96 (dt, *J* = 17.1, 1.3 Hz, 1H), 4.92 (dt, *J* = 10.4, 1.3 Hz, 1H ), 3.27 (dd, *J* = 13.9, 10.1 Hz, 1H), 3.24–3.15 (br, 1H), 3.09–2.98 (m, 1H), 2.80–2.69 (m, 2H), 2.36–2.27 (br, 1H), 1.77–1.68 (m, 2H), 1.68–1.58 (m, 1H), 1.45–1.36 (m, 1H), 1.17 (dd, *J* = 13.7, 6,7 Hz, 1H). ^13^C NMR (125 MHz, CDCl_3_): δ = 197.8, 150.2, 148.6, 146.0, 141.1, 140.5, 140.4, 136.0, 130.6, 129.2, 127.5, 127.3, 126.7, 126.3, 123.8, 119.5, 115.99, 114.9, 61.5, 58.6, 55.9, 41.0, 39.7, 27.9, 27.3, 26.1. HMRS (ASAP^+^) C_28_H_30_N_3_S [(M+H)^+^]: calculated: 440.2160, found: 440.2159. IR: 3256, 2937, 1480 cm^−1^.

## 5. Conclusions

In conclusion, this work reported a complete study to synthesize fluorinated dithioesters. After optimization, an unprecedented one-pot reaction was developed corresponding to a Grignard reaction—Mitsunobu type substitution. Subsequently, the reactivity of these fluorinated dithioesters was evaluated with several primary amines which led to excellent reactivity compared to usual dithioesters, i.e., the methyl dithioester or the S-thiobenzoylglycolic acid. For the trifluoroethyl dithioester **3c**, the addition of one equivalent of amine is sufficient to achieve a quasi-quantitative conversion in accordance with green chemistry. This work revealed the potential of the trifluoroethyl group to activate a dithioester function for the synthesis of a 4-styrenylthioamide cinchonidine monomer in a very short time. This new access to functionalized chiral thioamides is currently used in our laboratory for an application in polymer-supported organocatalysis.

## Figures and Tables

**Table 1:** Substrate scopes of dithioesters ^a^.

Entry	Dithioacids	R^1^	R^2^	Yield (%) ^b^	Dithioesters
1	**1a**	H	CH_3_	68	**2a**
2		H	CH_2_CH_3_	72	**2b**
3		H	CH_2_CF_3_	72	**2c**
4		H	CH_2_CH_2_CF_3_	78	**2d**
5	**1b**	vinyl	CH_3_	35	**3a**
6		vinyl	CH_2_CH_3_	49	**3b**
7		vinyl	CH_2_CF_3_	21	**3c**
8		vinyl	CH_2_CH_2_CF_3_	53	**3d**

^a^ All reactions were carried out with 1 equiv of dithiocarboxylic acid in acetone at room temperature for 3 h. ^b^ Isolated yields after purification by column chromatography.

**Table 2:** Mitsunobu-type substitution from organomagnesium reagents.

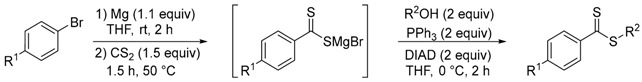				
Entry	R^1^	R^2^	Yield (%)	Dithioesters
1	H	CH_2_CH_3_	73	**2b**
2	H	CH_2_CF_3_	71	**2c**
3	H	CH_2_Ph	59	**2e**
4	vinyl	CH_2_CF_3_	40	**3c**

## Data Availability

The data presented in this study are available in the Supplementary Material.

## Supplementary Materials

The following supporting information can be downloaded at: https://www.mdpi.com/article/10.3390/molecules28217333/s1. Methyl dithiobenzoate **2a**;Ethyl dithiobenzoate **2b**; 2,2,2-trifluoroethyl dithiobenzoate **2c**;2,2,2-trifluoropropyl dithiobenzoate **2d**; Benzyl dithiobenzoate **2e**; Methyl 4-vinyldithiobenzoate **3a**; 2,2,2-trifluoroethyl 4-vinyldithiobenzoate **3c**; 2,2,2-trifluoropropyl 4-vinyldithiobenzoate **3d**; 2,2,2-trifluoroethyl 4-vinylthiobenzoate **4**; *N*-benzyl-4-vinylbenzothioamide **5a**; 4-vinyl-*N*-propan-2-ylbenzothioamide **5b**; 4-vinyl-*N*-(1-phenylethyl)benzothioamide **5c**; *N*-(9-Deoxyepicinchonidin-9-yl)-4-vinylbenzothioamide **5d**.

## Sample Availability

Samples of the compounds are not available from the authors.
