# Supplementary material for: Synthesis and Reactivity of Fluorinated Dithiocarboxylates to Prepare Thioamides—Effective Access to a 4-Styrenylthioamide-Cinchona Alkaloid Monomer
Source: Molecules. 2023 Oct 30;28(21):7333. doi: 10.3390/molecules28217333 (PMC10649591; doi:10.3390/molecules28217333)

Supporting Information

Synthesis and reactivity of fluorinated dithiocarboxylates to prepare thioamides. Effective access to a 4-styrenylthioamide-cinchona alkaloid monomer.

Aimar Gonzalo-Barquero[1], Bénédicte Lepoittevin[1], Jacques Rouden[1] and Jérôme Baudoux[1,*]

[1] Normandie Université, Laboratoire de Chimie Moléculaire et Thioorganique, UMR 6507, ENSICAEN, UNICAEN, CNRS, 6 Bd du Maréchal Juin, 14050 Caen, France

* Correspondence: jerome.baudoux@ensicaen.fr

Methyl dithiobenzoate 2a

^{1}H NMR

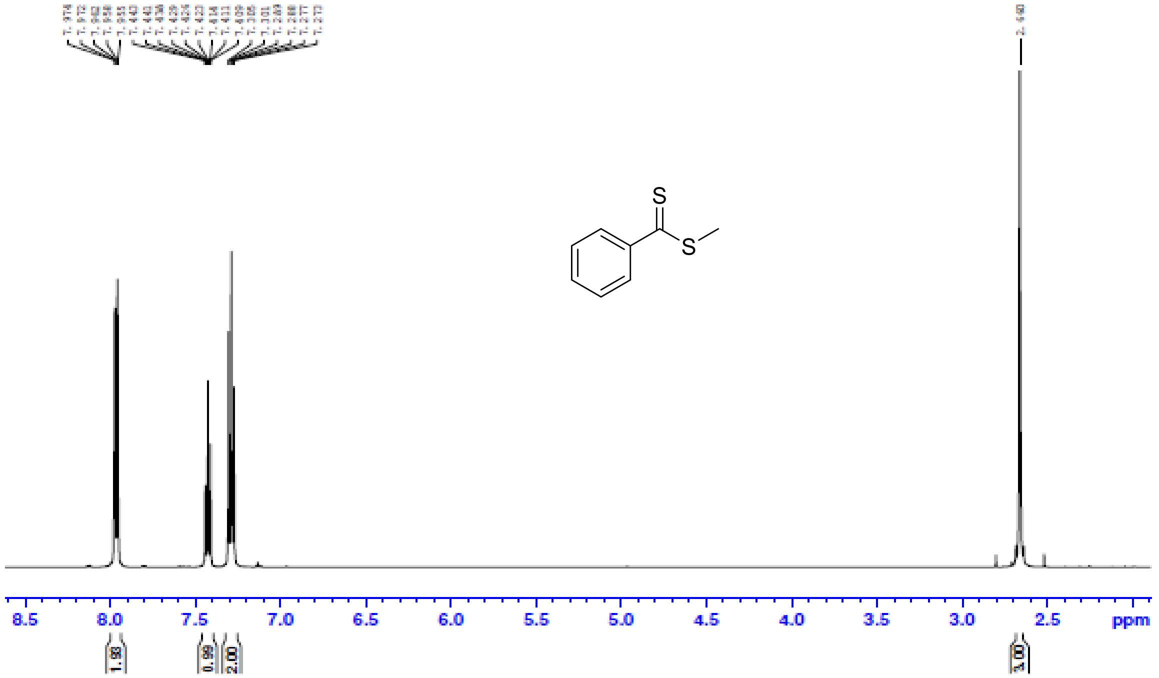

^{13}C NMR

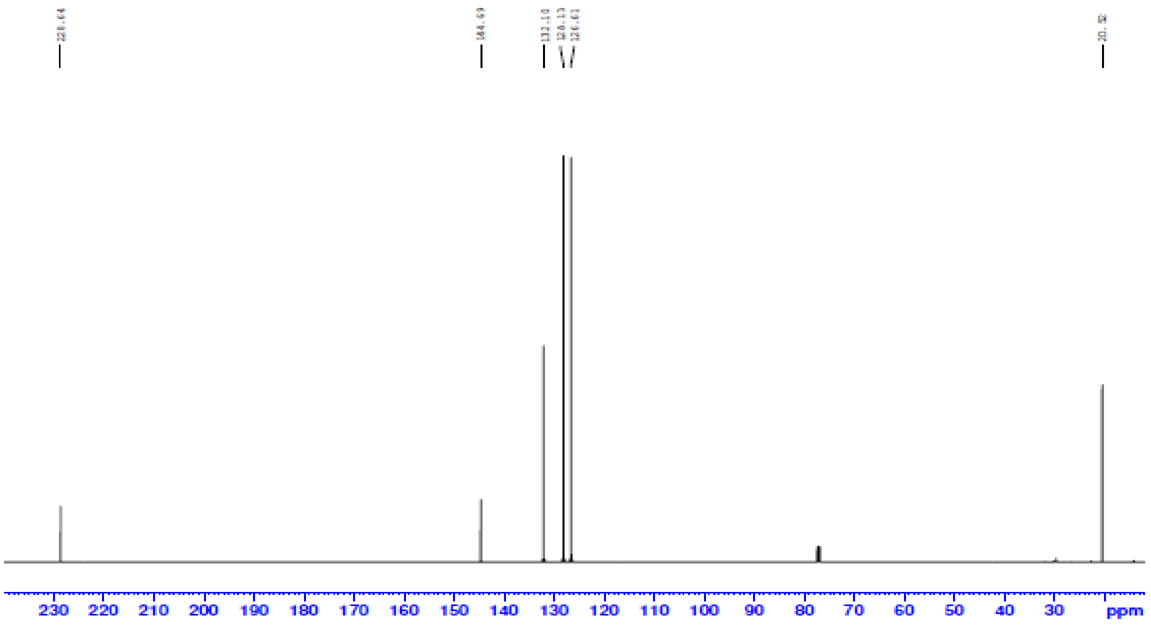

Ethyl dithiobenzoate 2b

^{1}H NMR

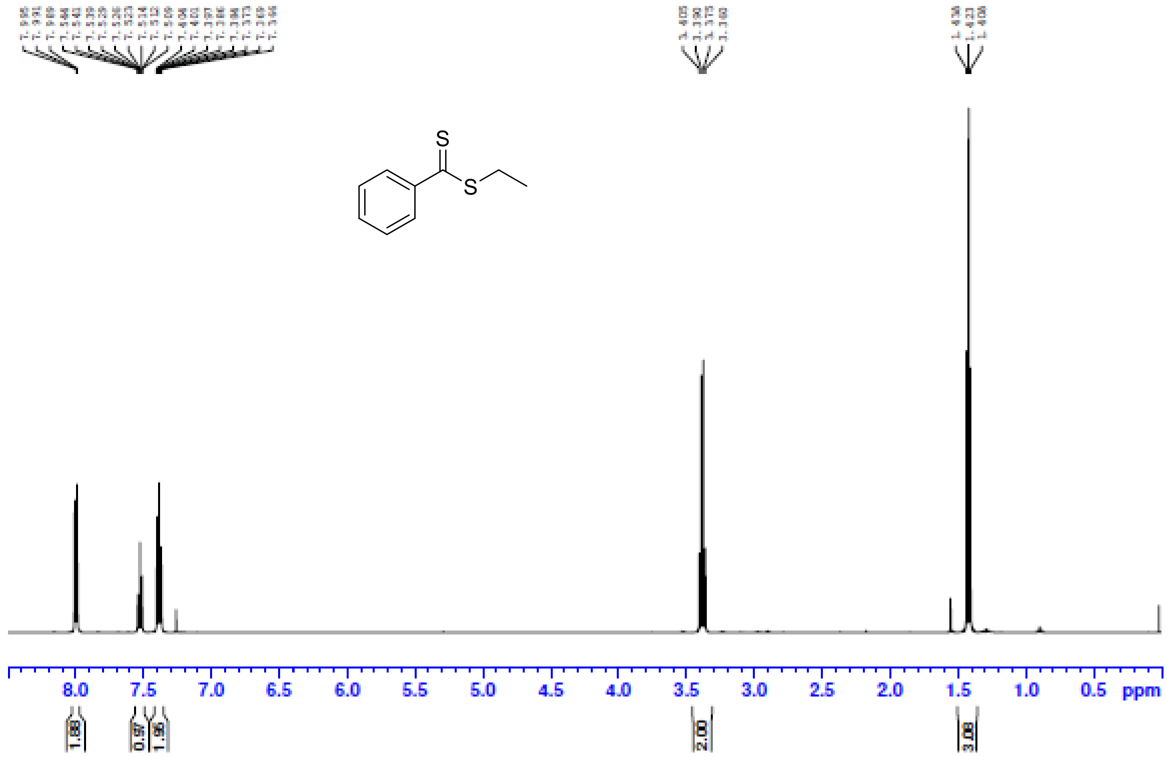

^{13}C NMR

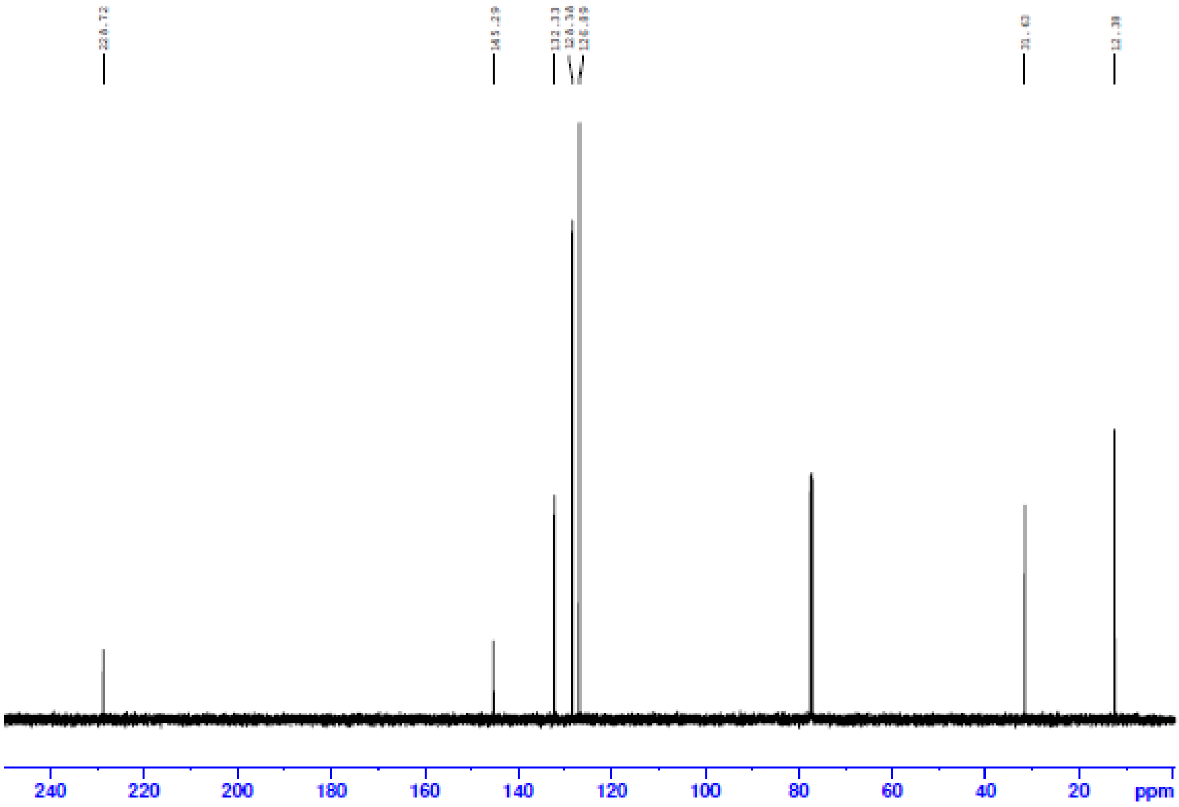

2,2,2-trifluoroethyl dithiobenzoate 2c

^{1}H NMR

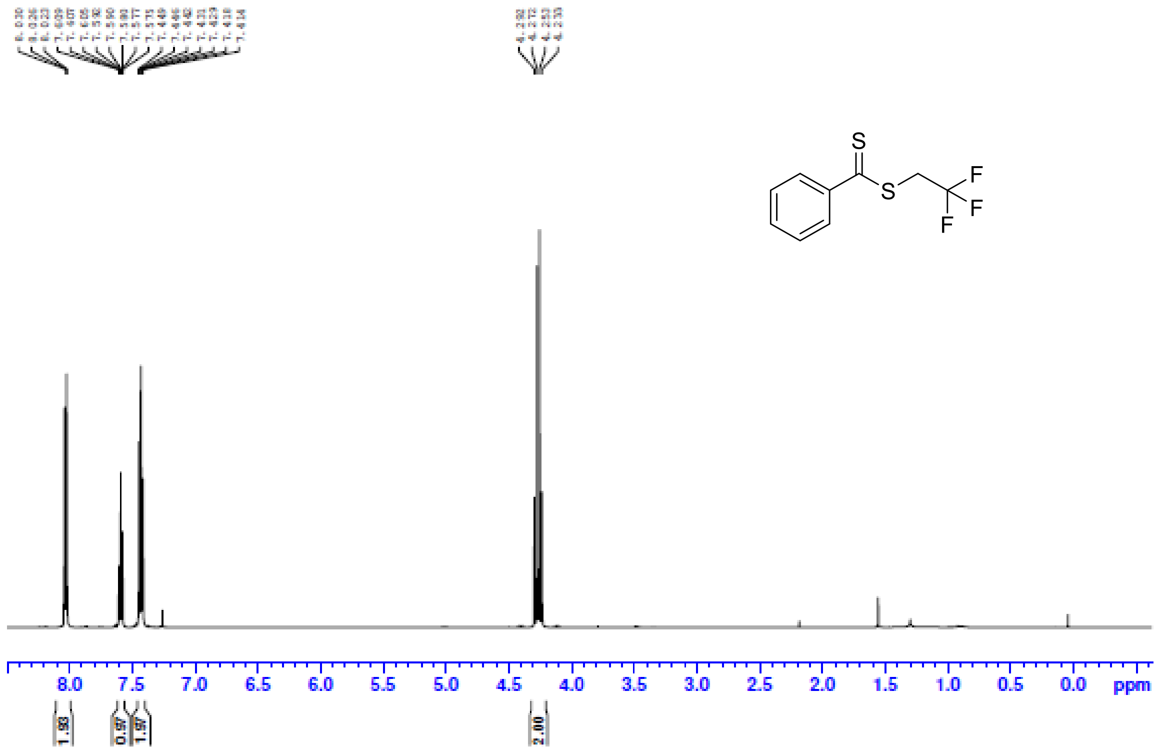

Supporting Information

^{13}C NMR

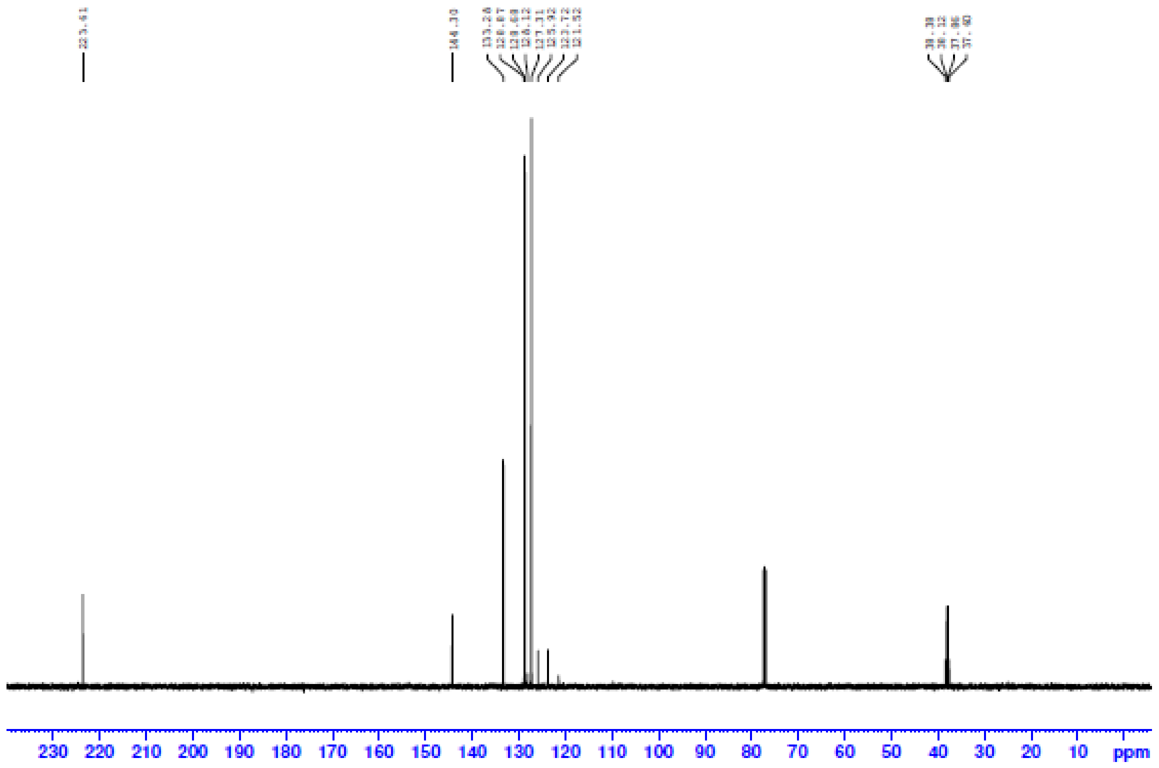

^{19}F NMR

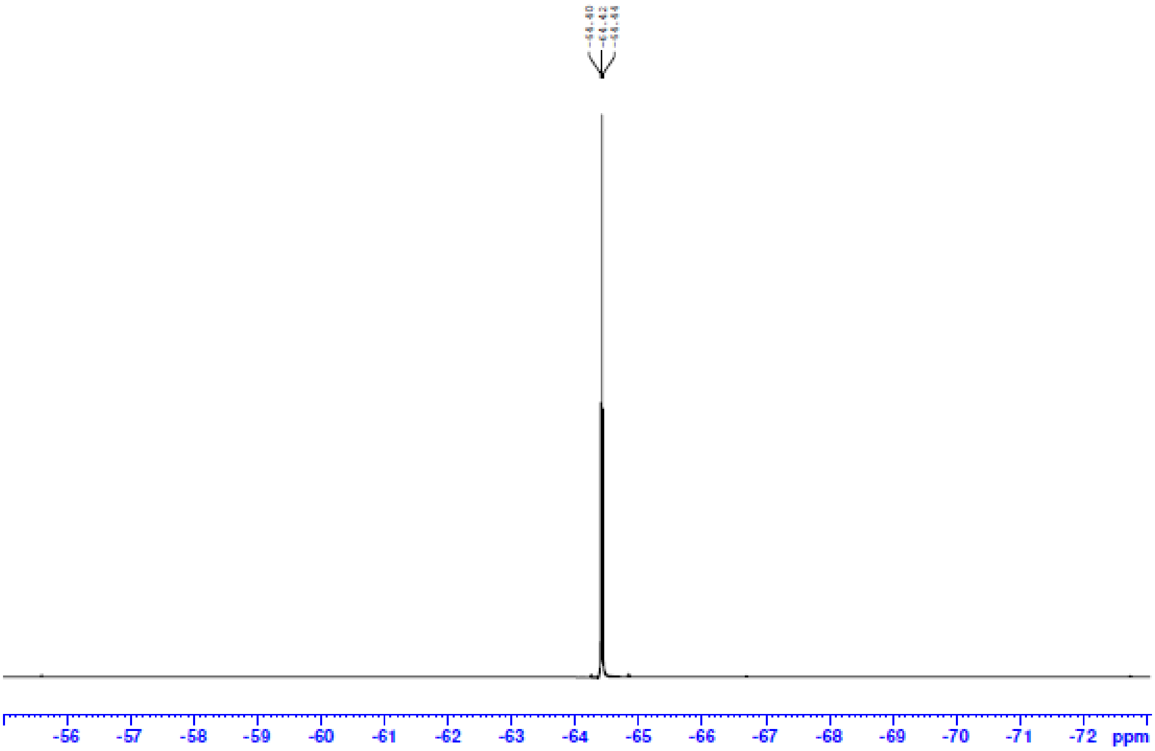

2,2,2-trifluoropropyl dithiobenzoate 2d

^{1}H NMR

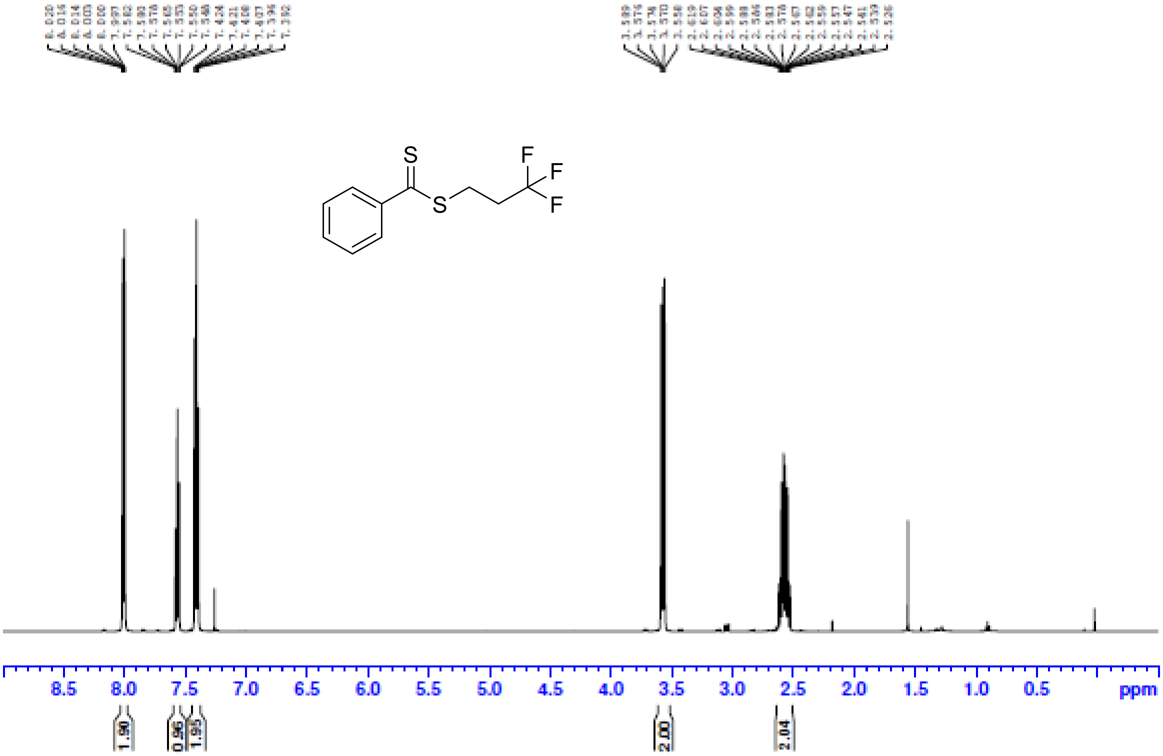

Supporting Information

^{13}C NMR

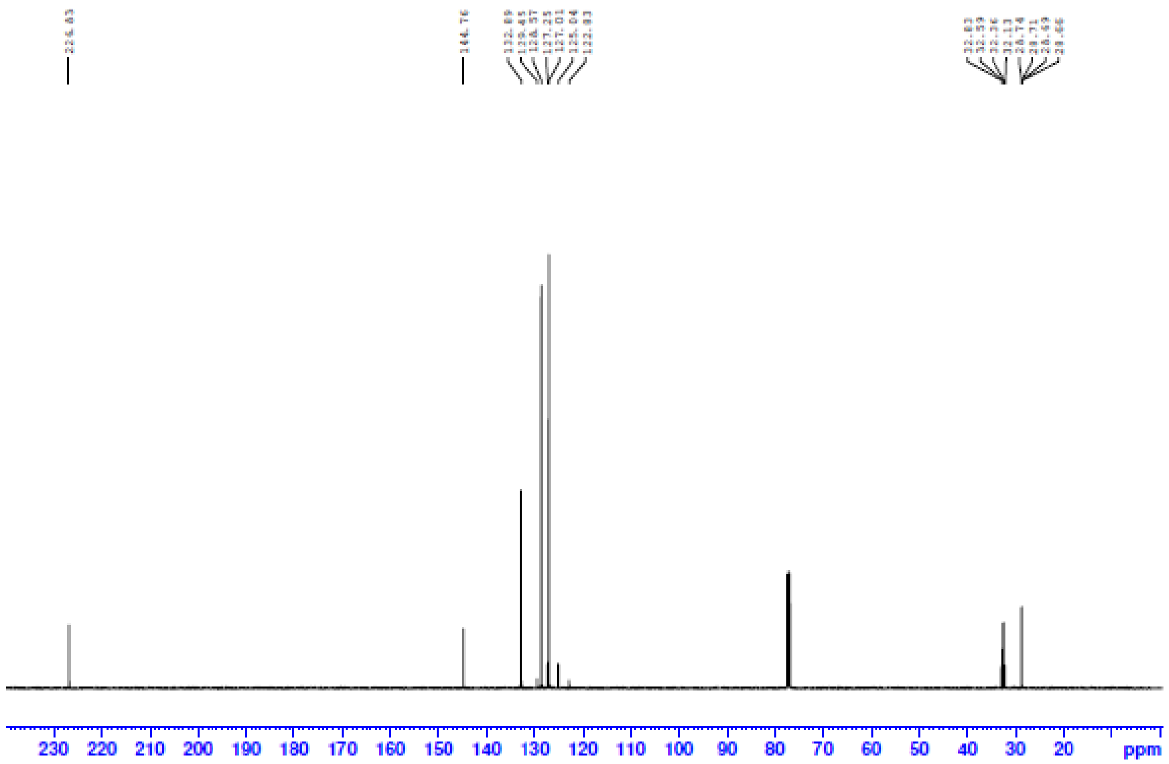

^{19}F NMR

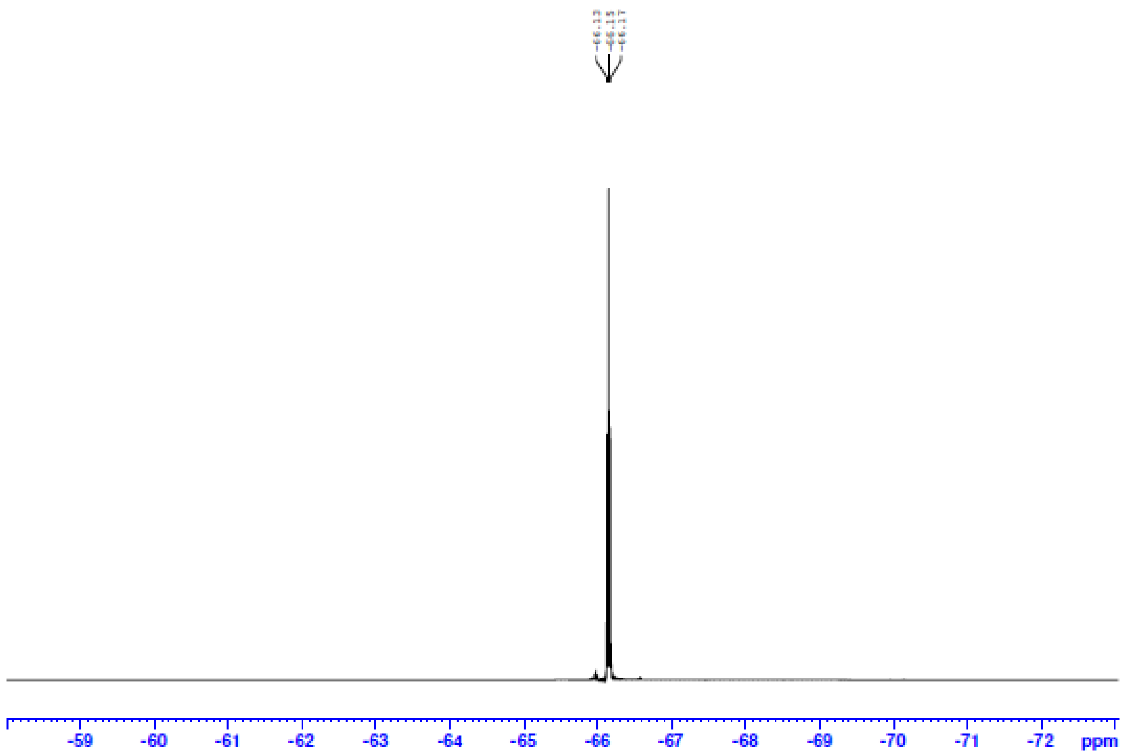

Supporting Information

Benzyl dithiobenzoate 2e

^{1}H NMR

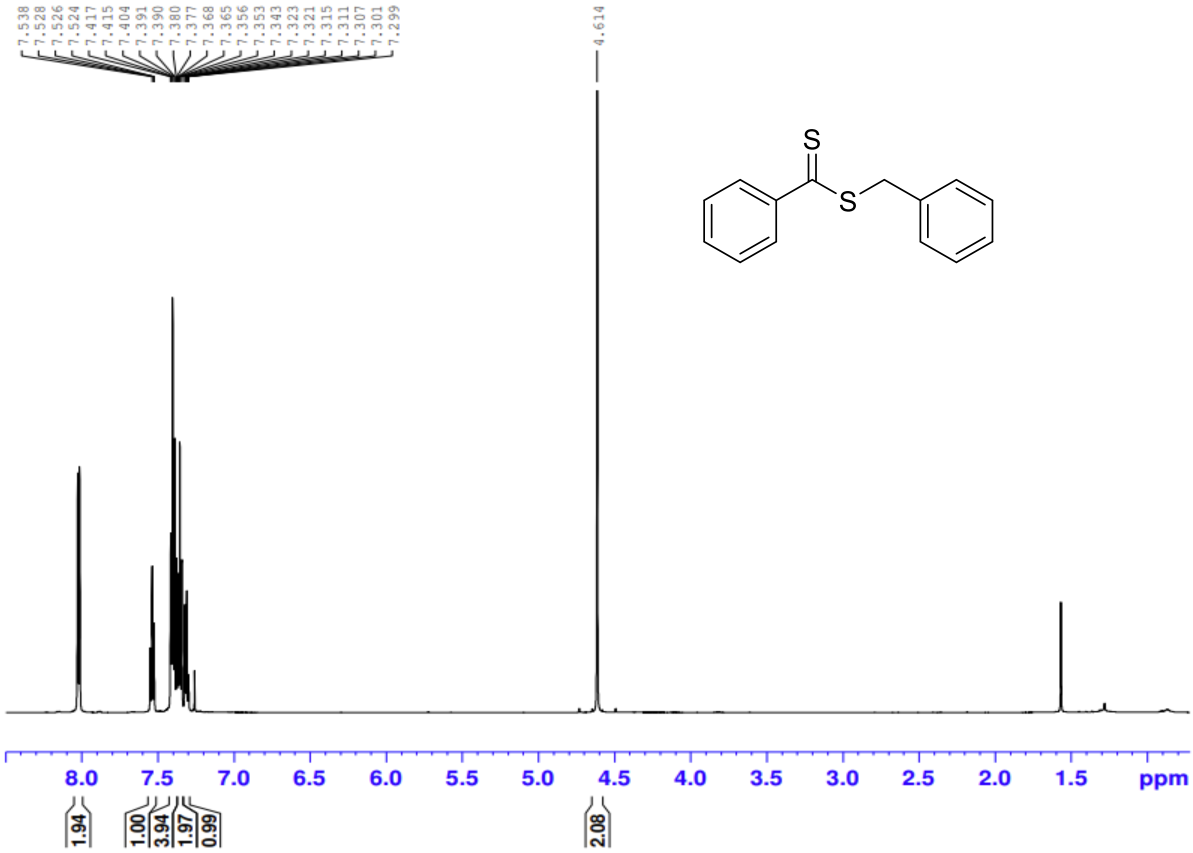

^{13}CNMR

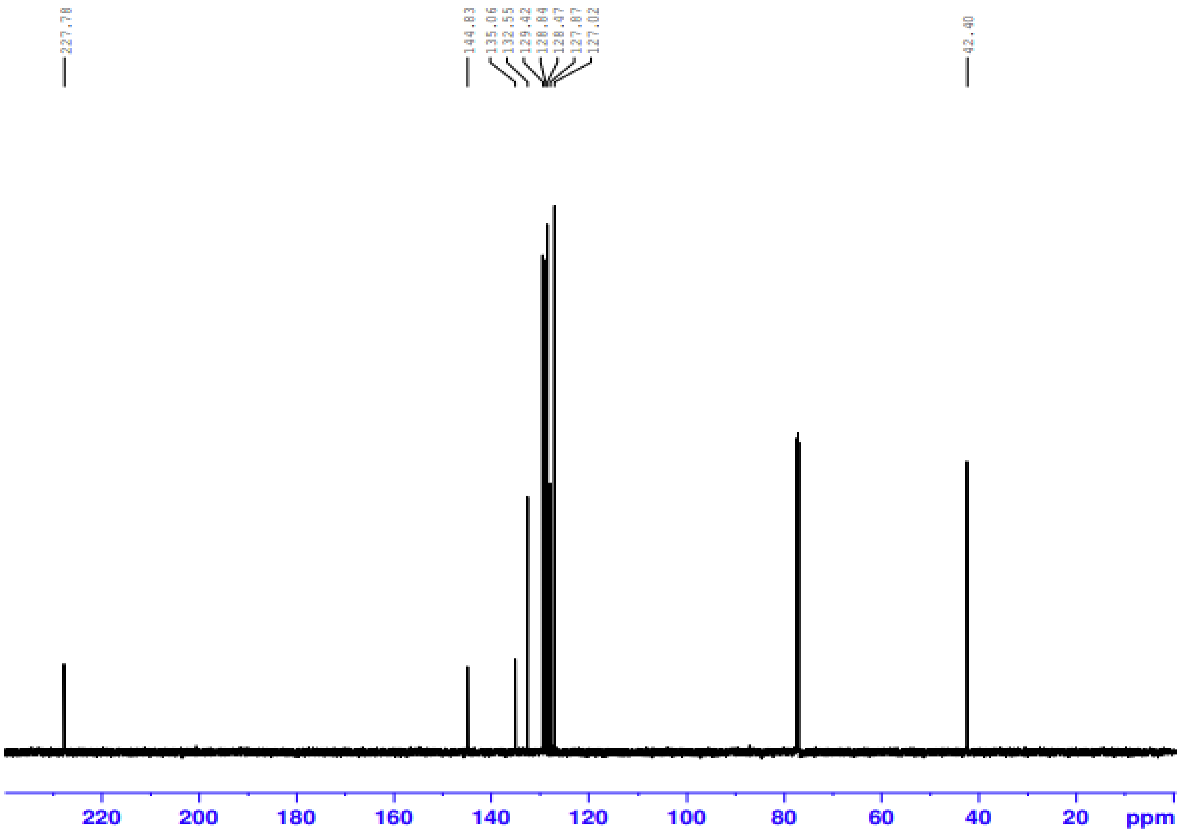

Methyl 4-vinyldithiobenzoate 3a

^{1}H NMR

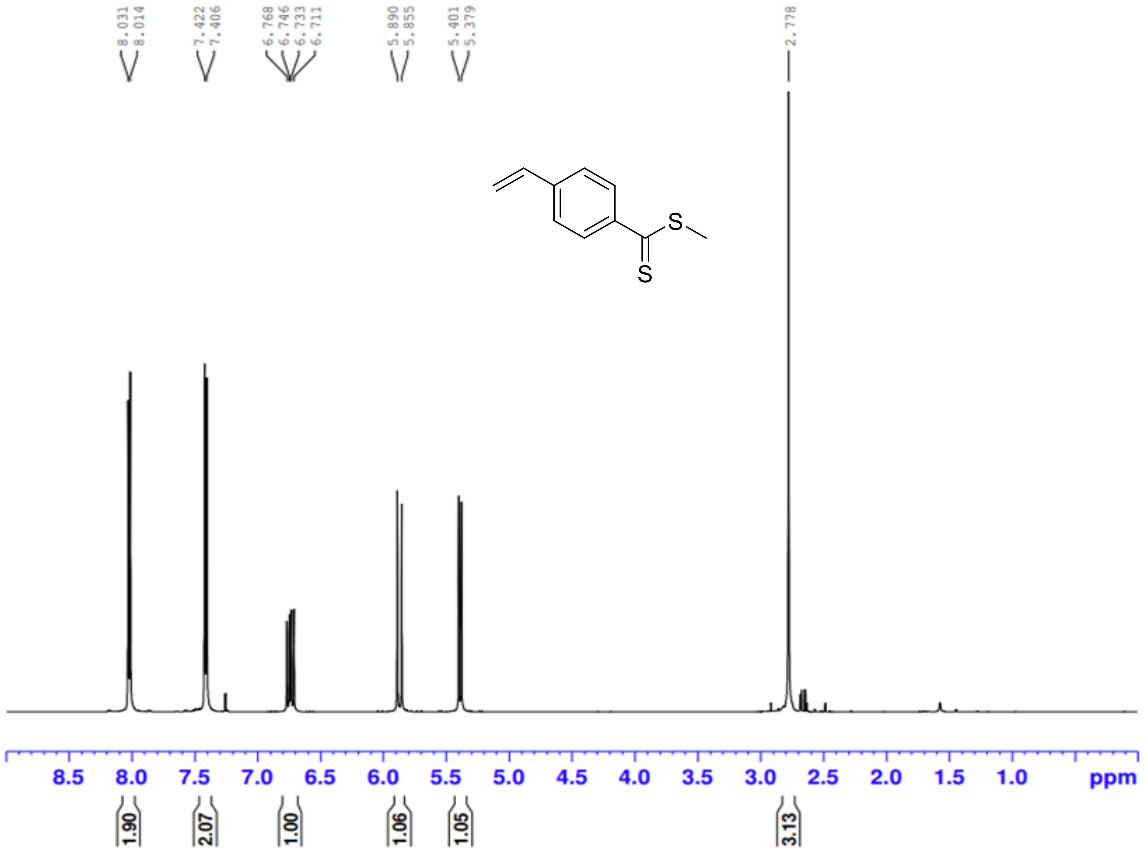

^{13}C NMR

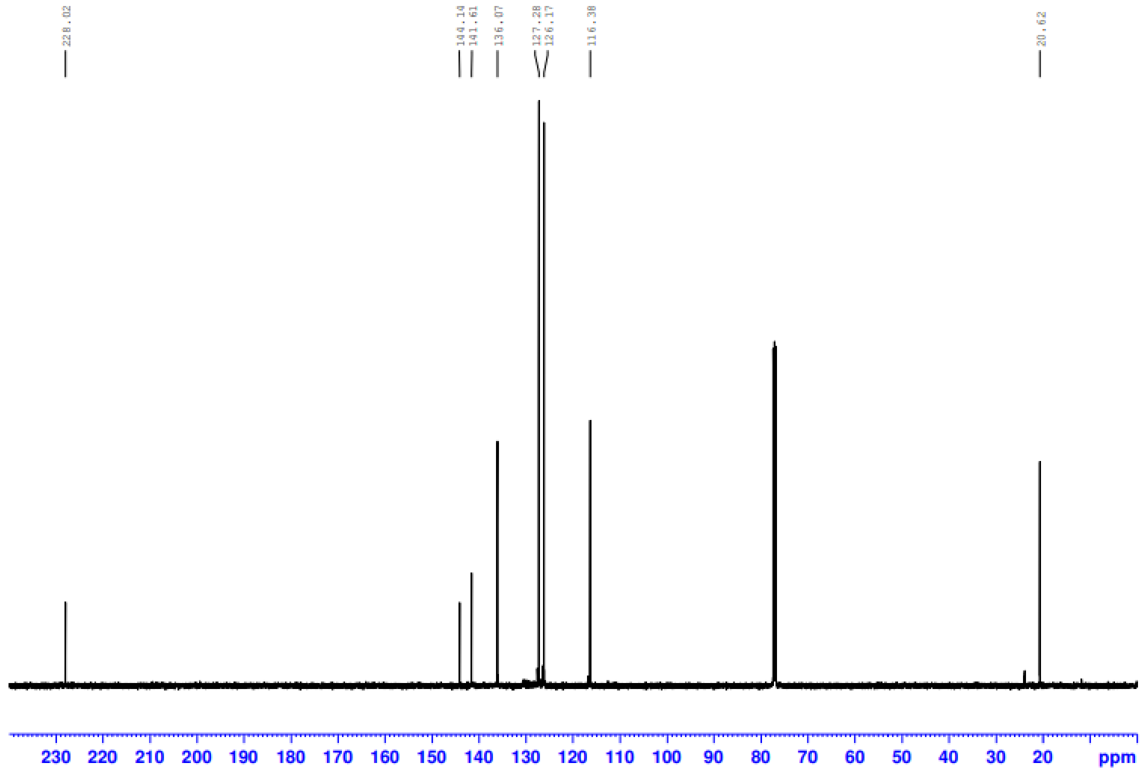

Ethyl 4-vinyldithiobenzoate 3b

Supporting Information

^{1}H NMR

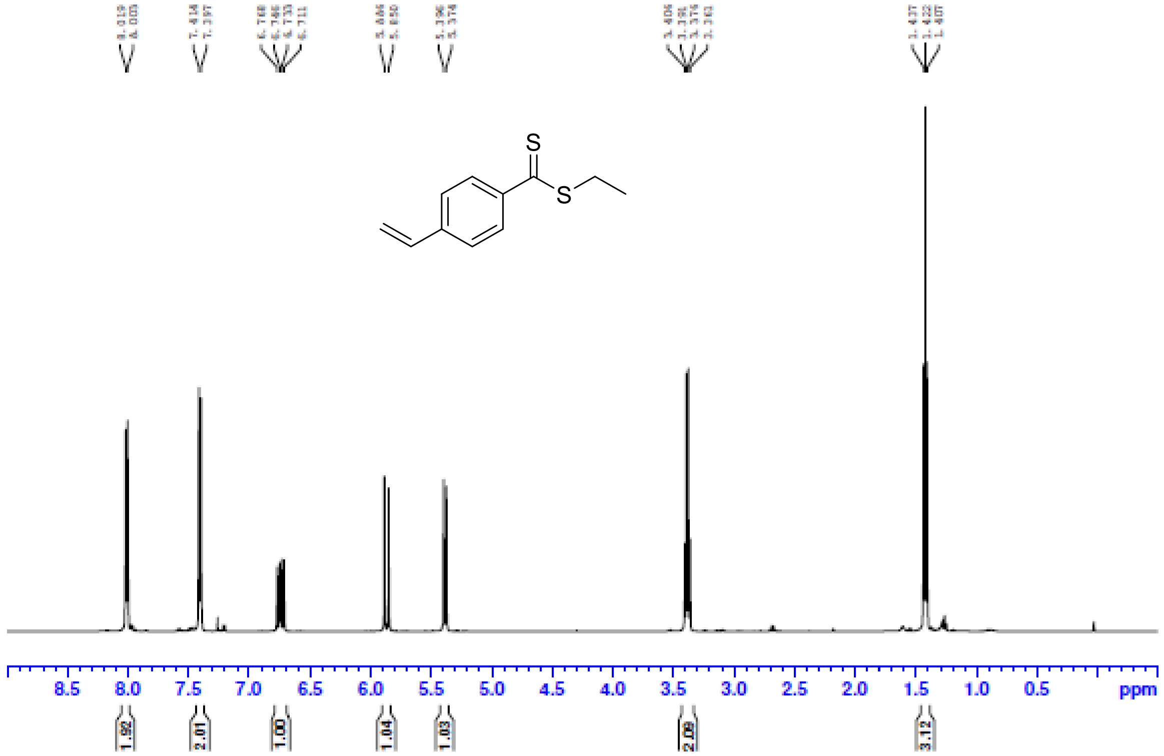

^{13}C NMR

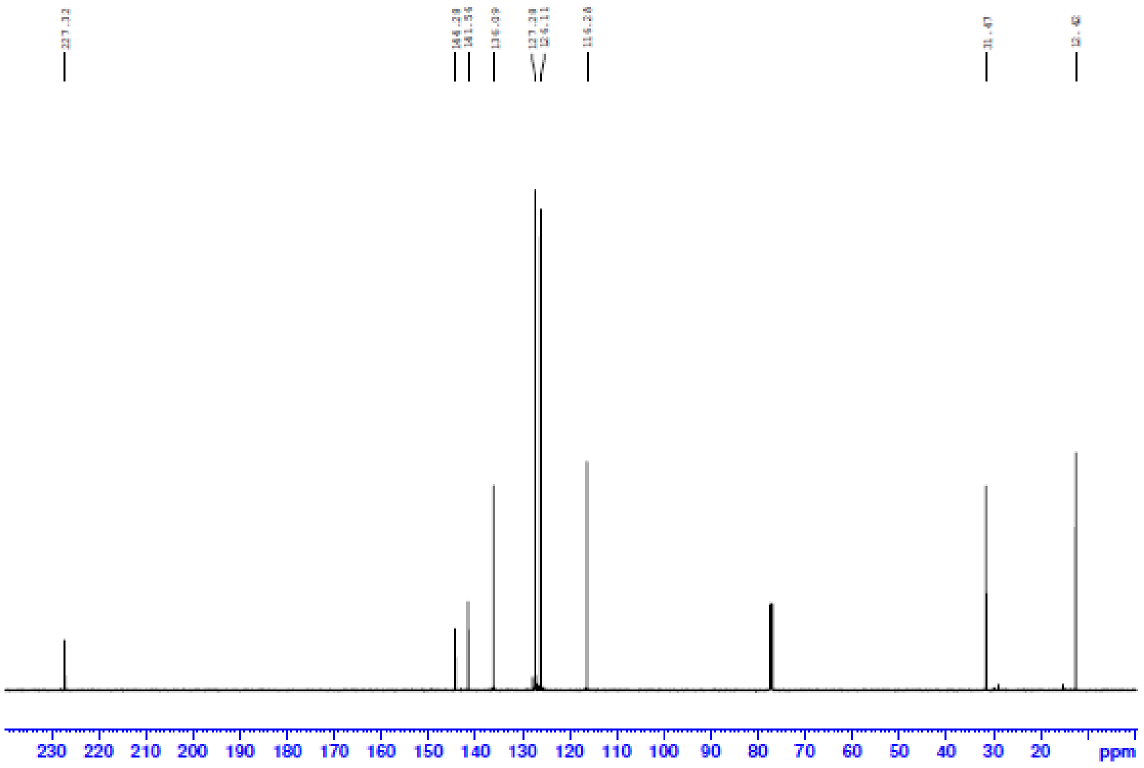

Supporting Information

2,2,2-trifluoroethyl 4-vinyldithiobenzoate 3c

^{1}H NMR

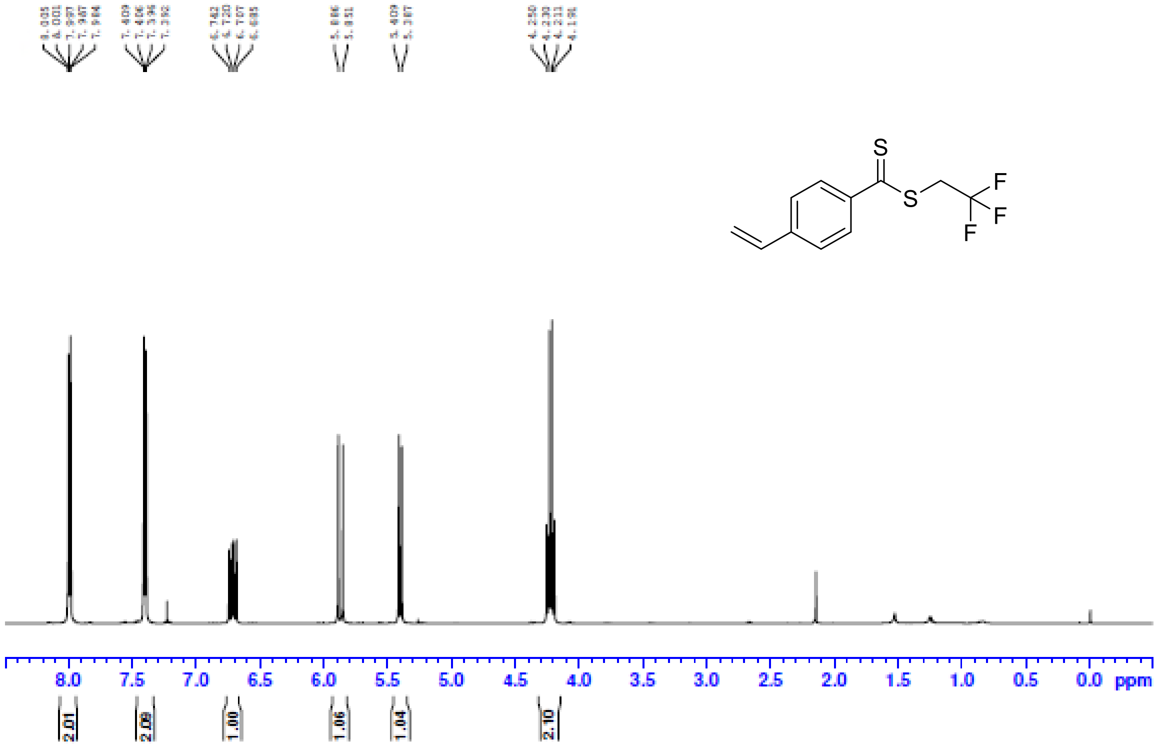

^{13}C NMR

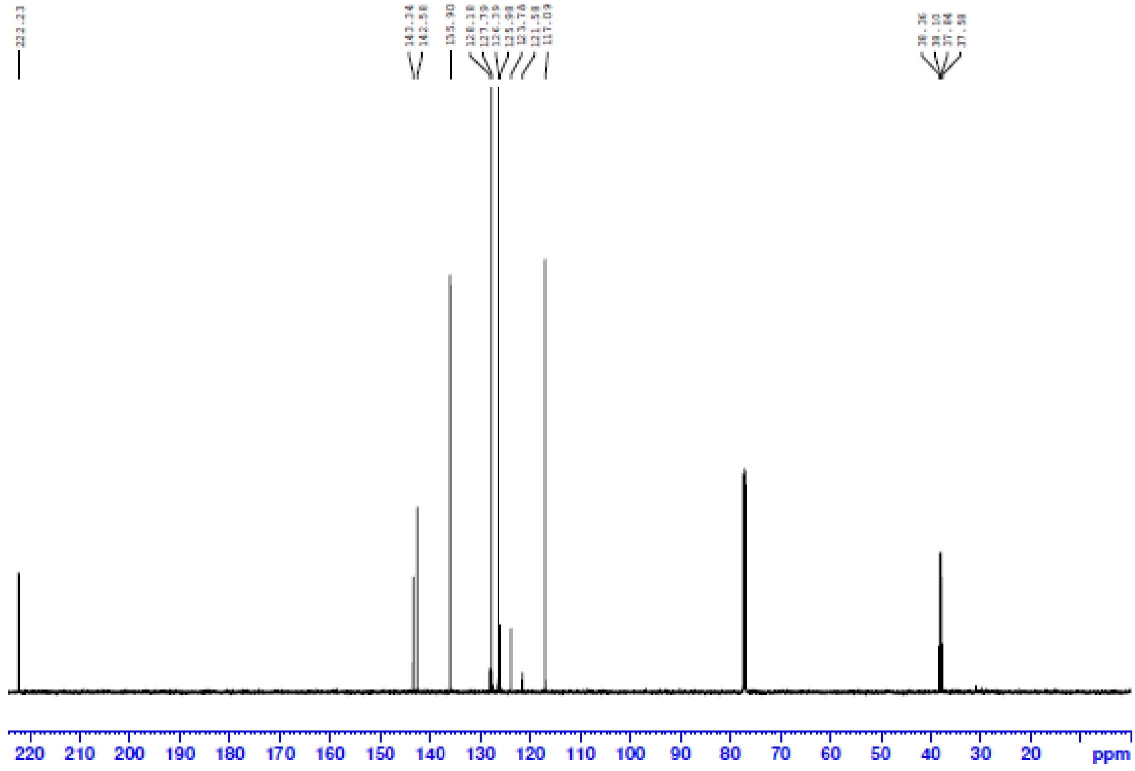

Supporting Information

^{19}F NMR

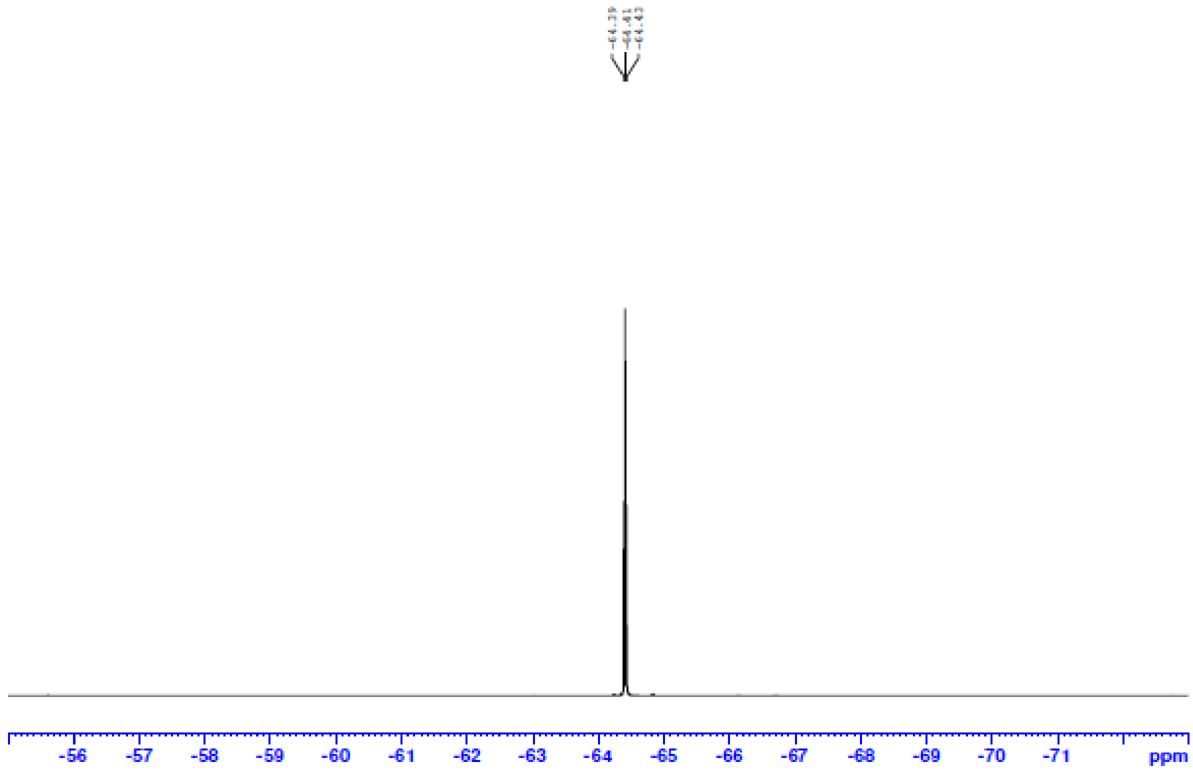

2,2,2-trifluoropropyl 4-vinyldithiobenzoate 3d

^{1}H NMR

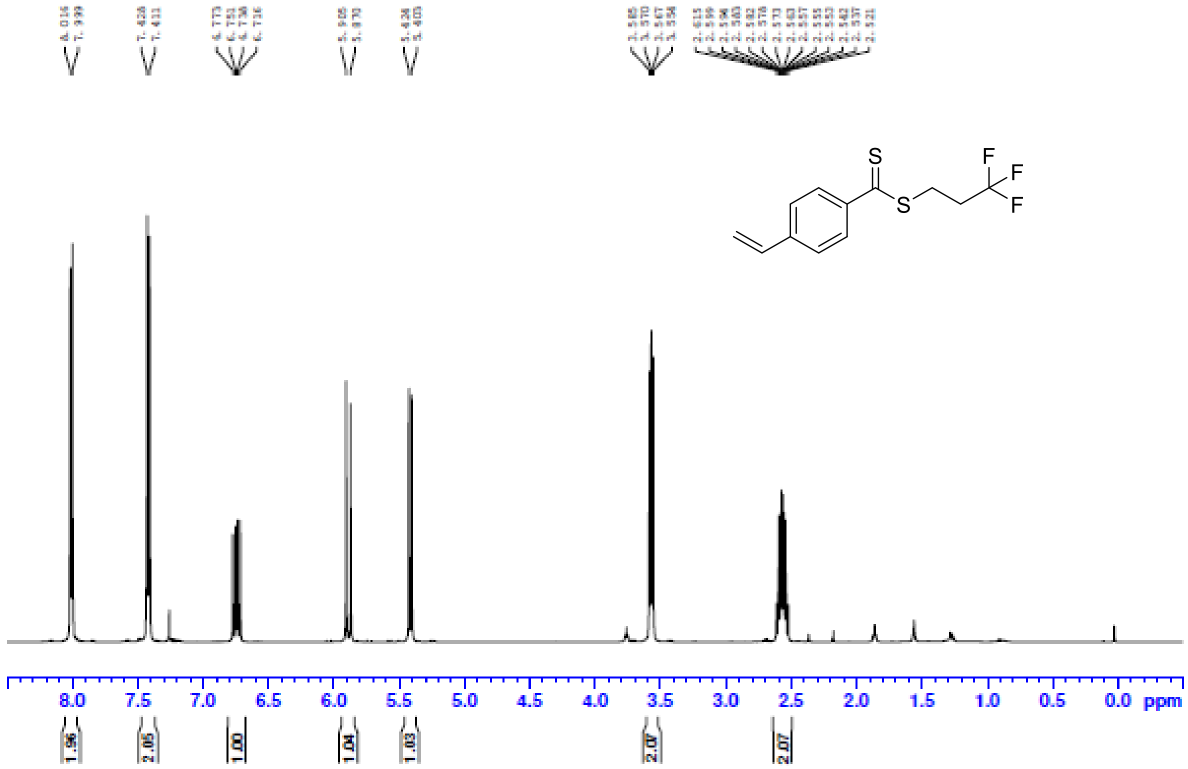

Supporting Information

^{13}C NMR

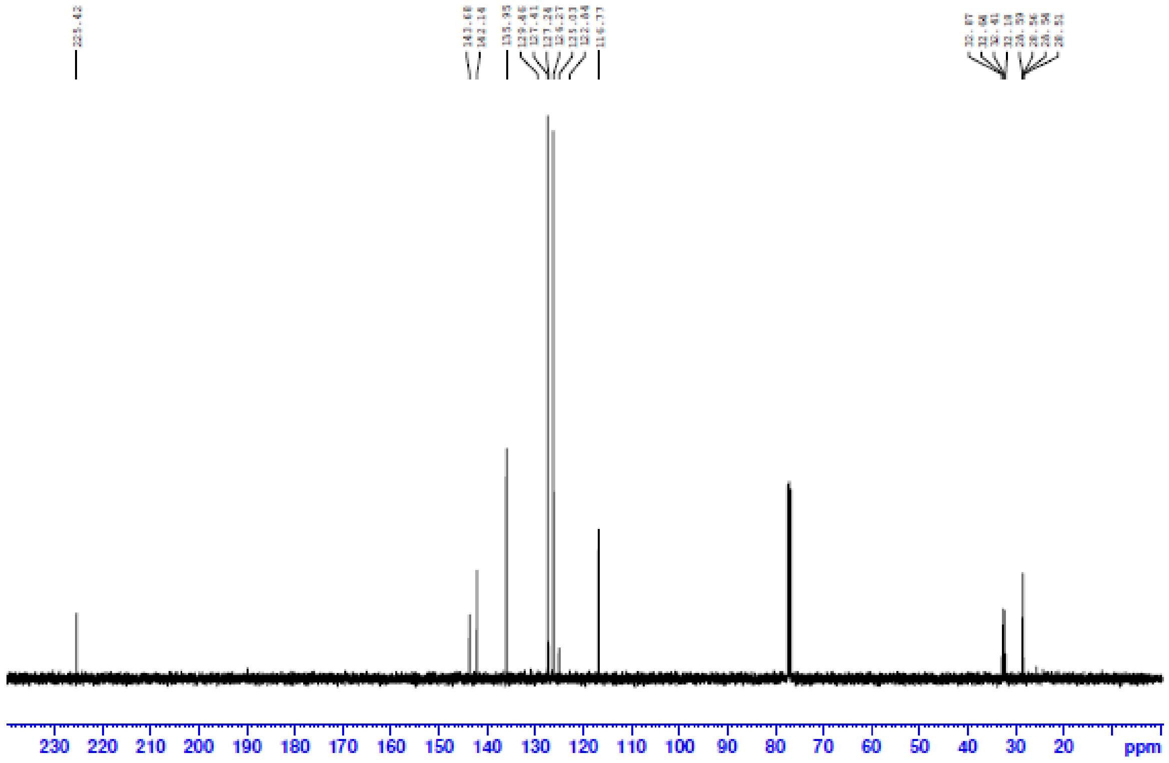

^{19}F NMR

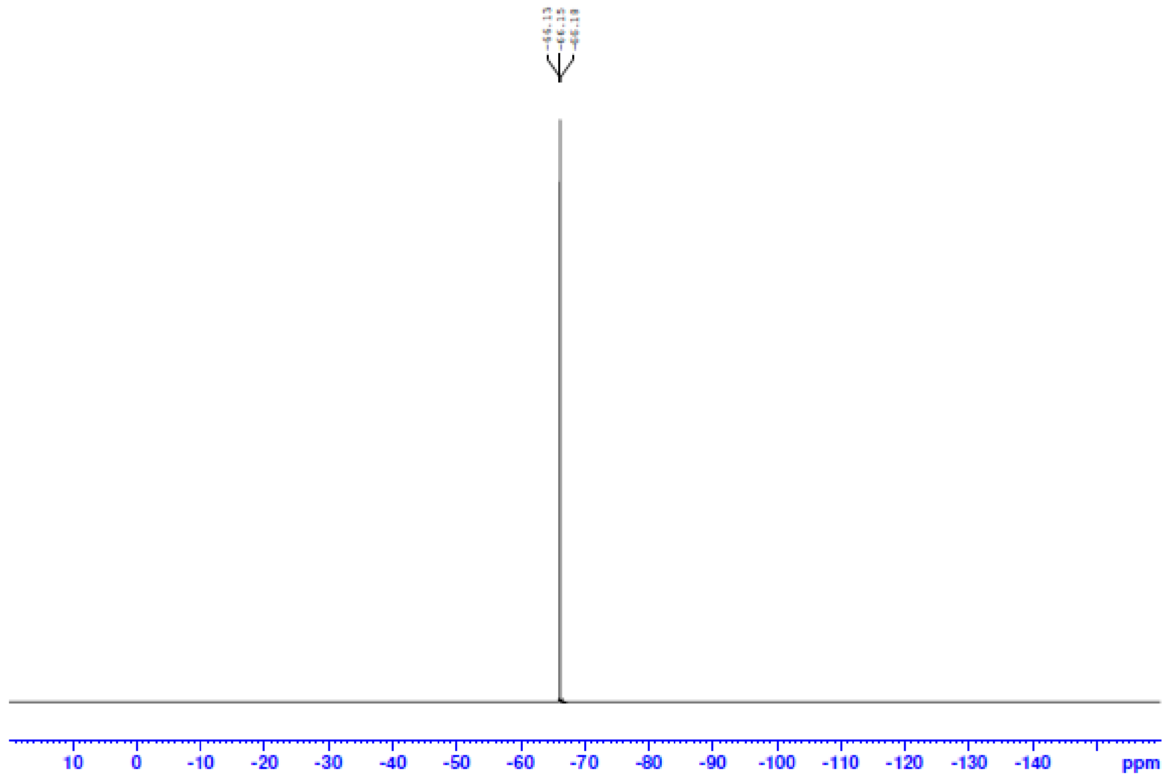

Supporting Information

2,2,2-trifluoroethyl 4-vinylthiobenzoate 4

^{1}H NMR

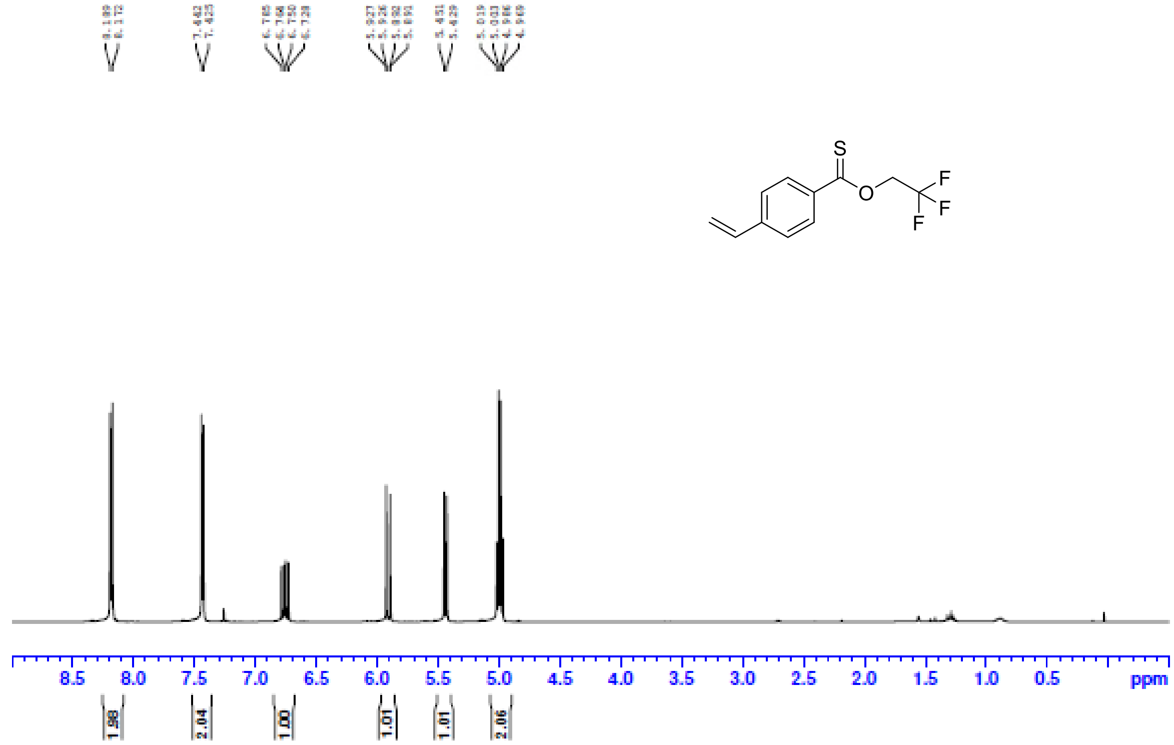

^{13}C NMR

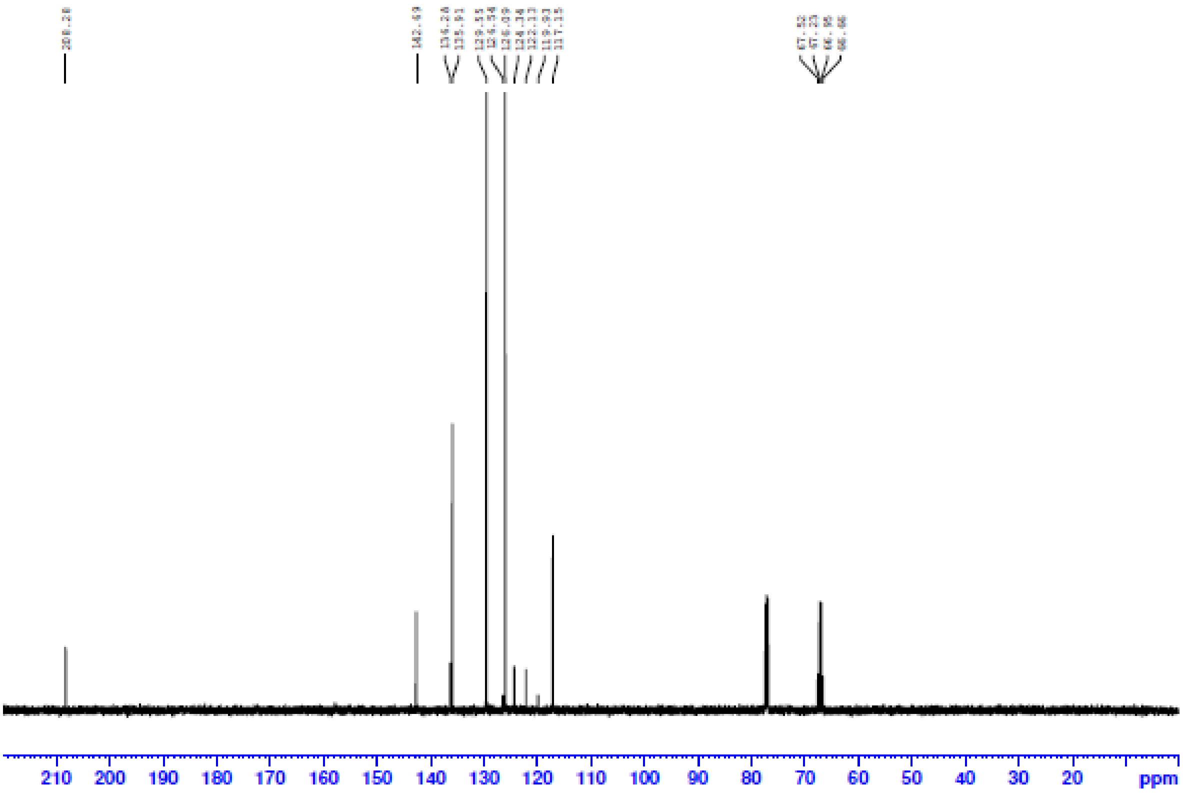
210 200 190 180 170 160 150 140 130 120 110 100 90 80 70 60 50 40 30 20 ppm

^{19}F NMR

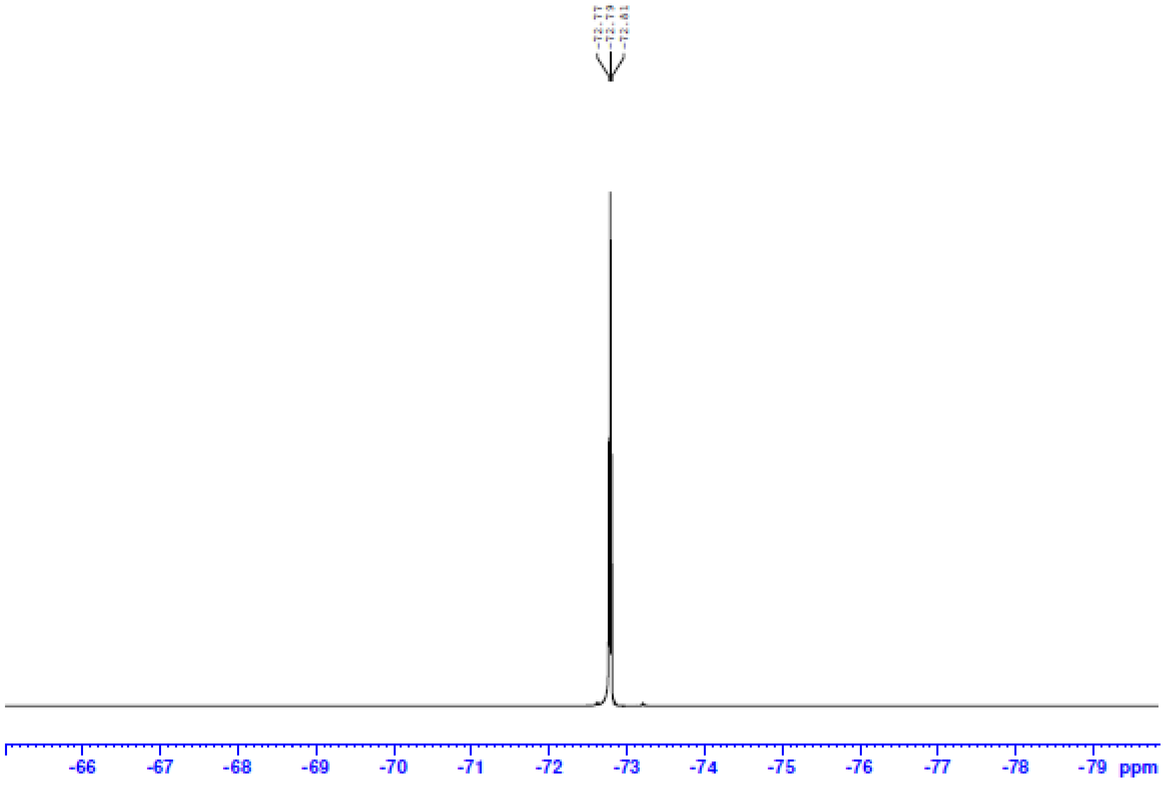
-66 -67 -68 -69 -70 -71 -72 -73 -74 -75 -76 -77 -78 -79 ppm

Supporting Information

***N*-benzyl-4-vinylbenzothioamide 5a**

^{1}H NMR

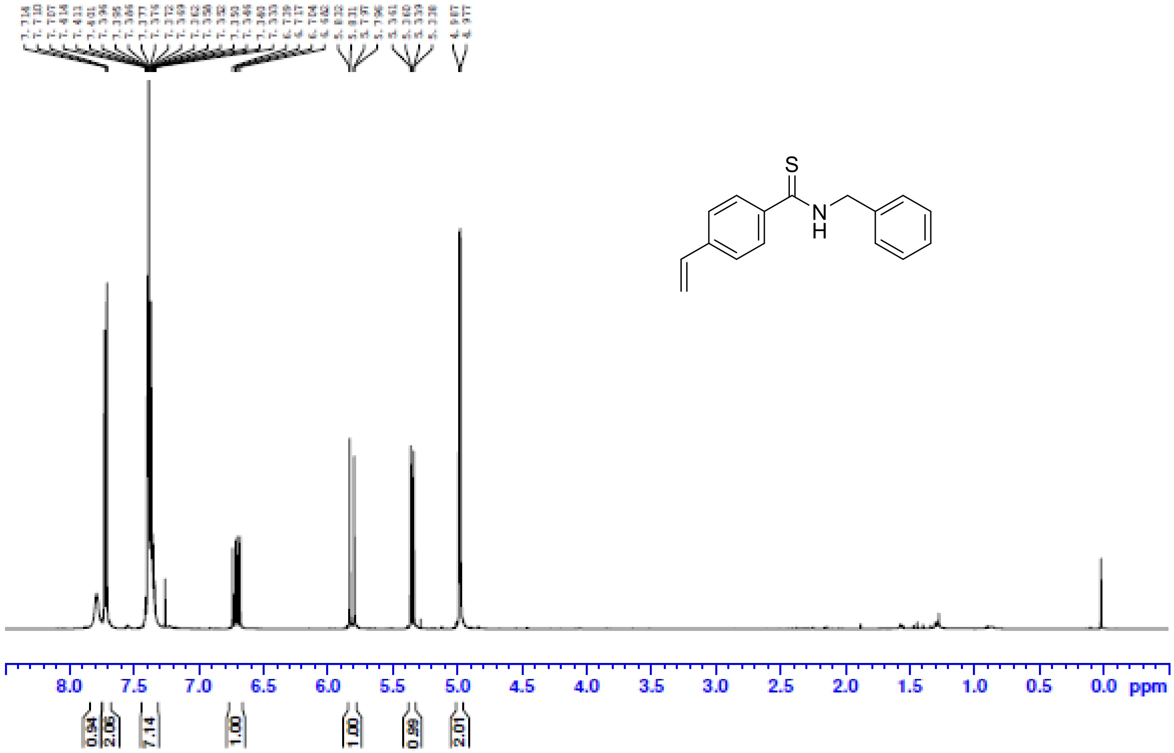

^{13}C NMR

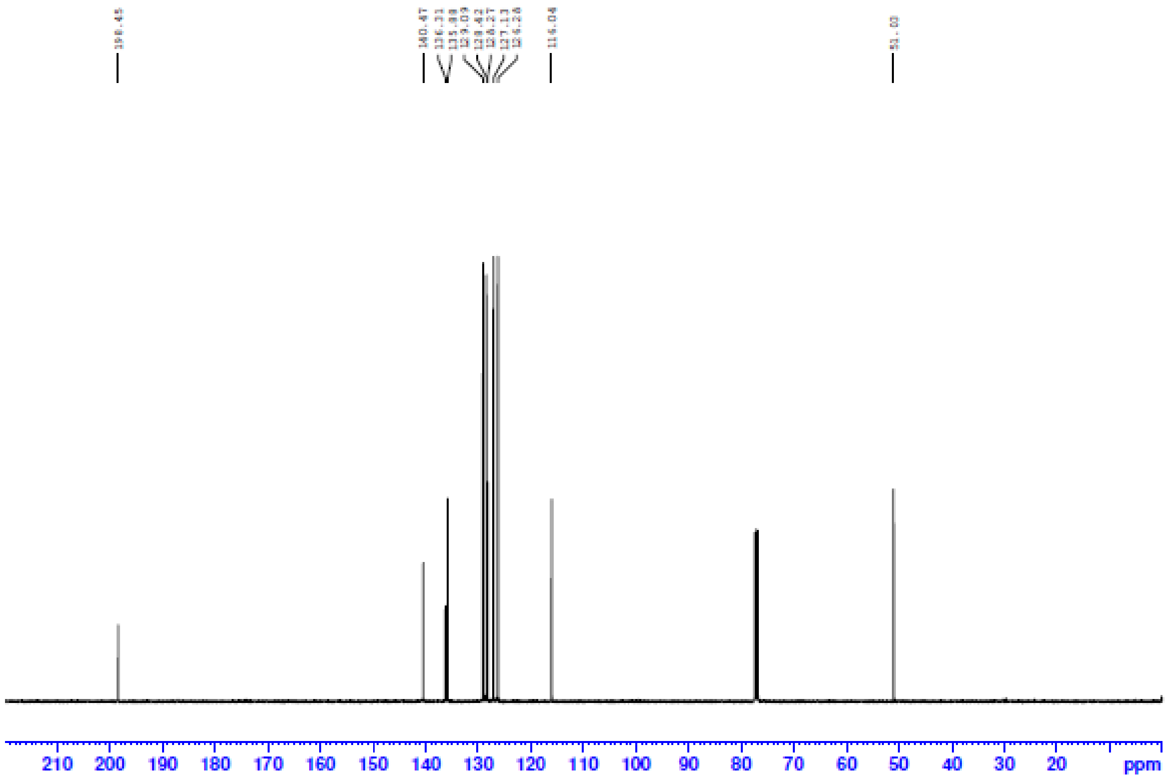

4-vinyl-*N*-propan-2-ylbenzothioamide 5b

^{1}H NMR

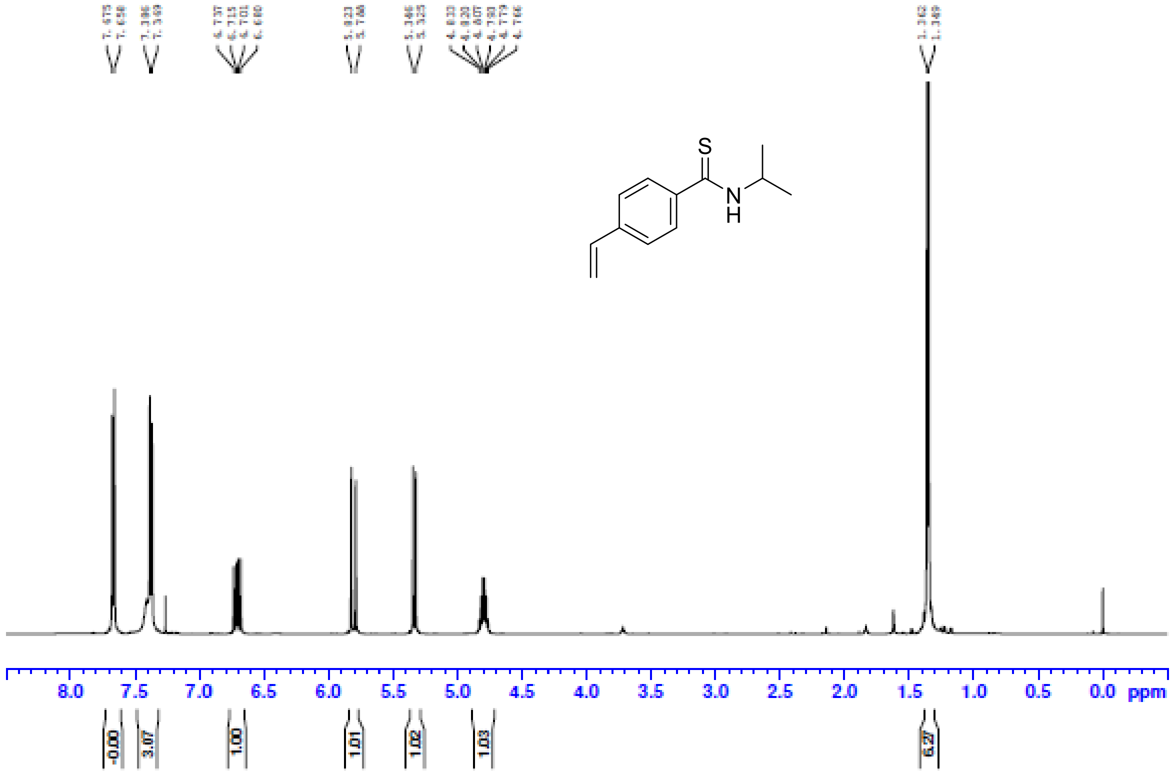

Supporting Information

^{13}C NMR

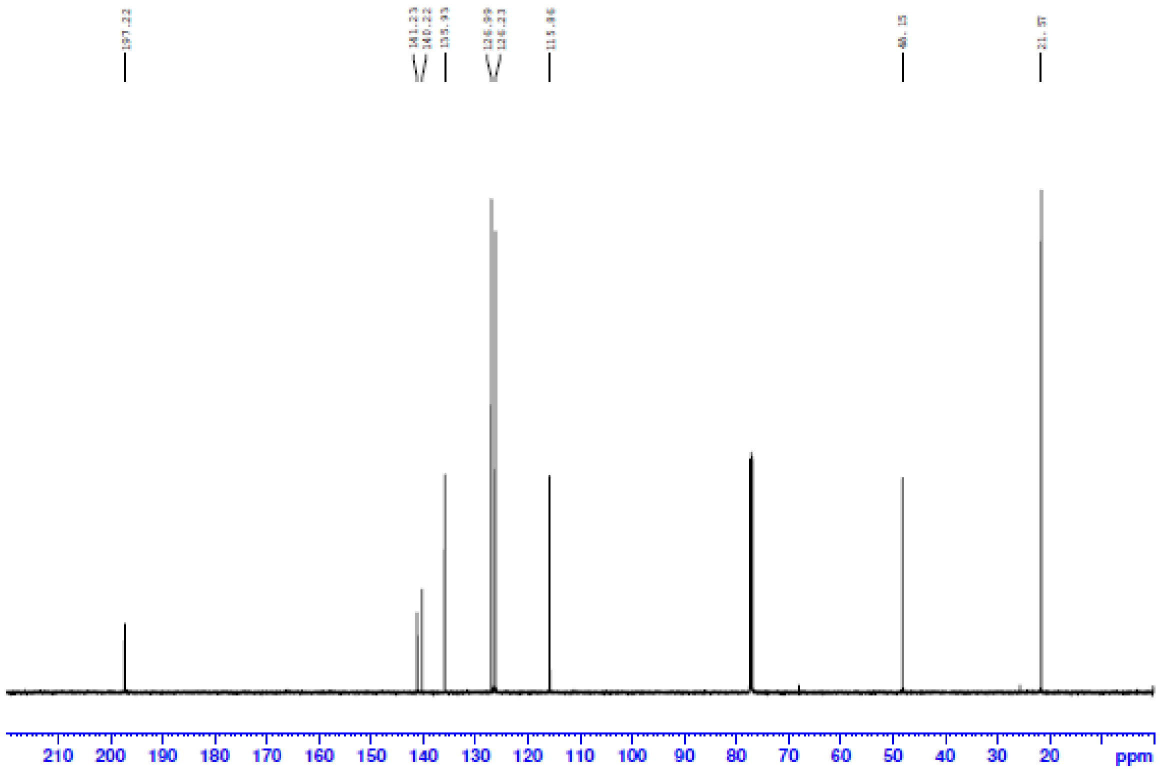

4-vinyl-*N*-(1-phenylethyl)benzothioamide 5c

^{1}H NMR

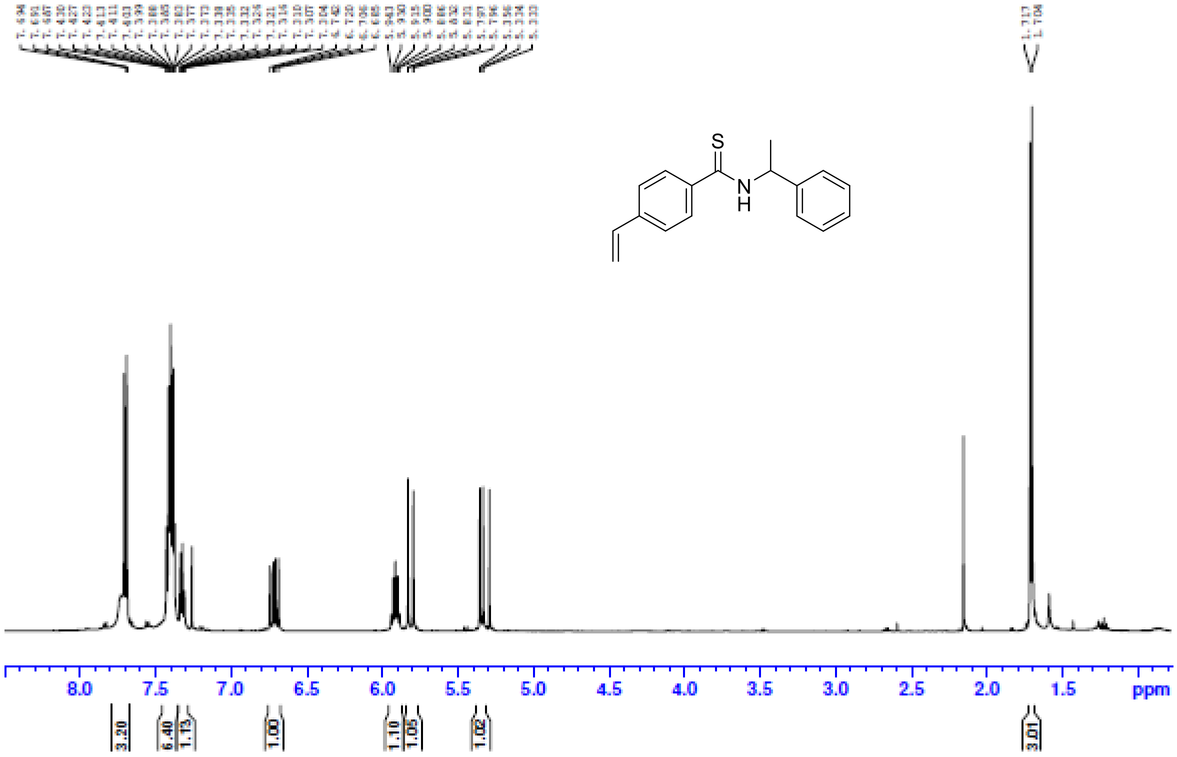

^{13}C NMR

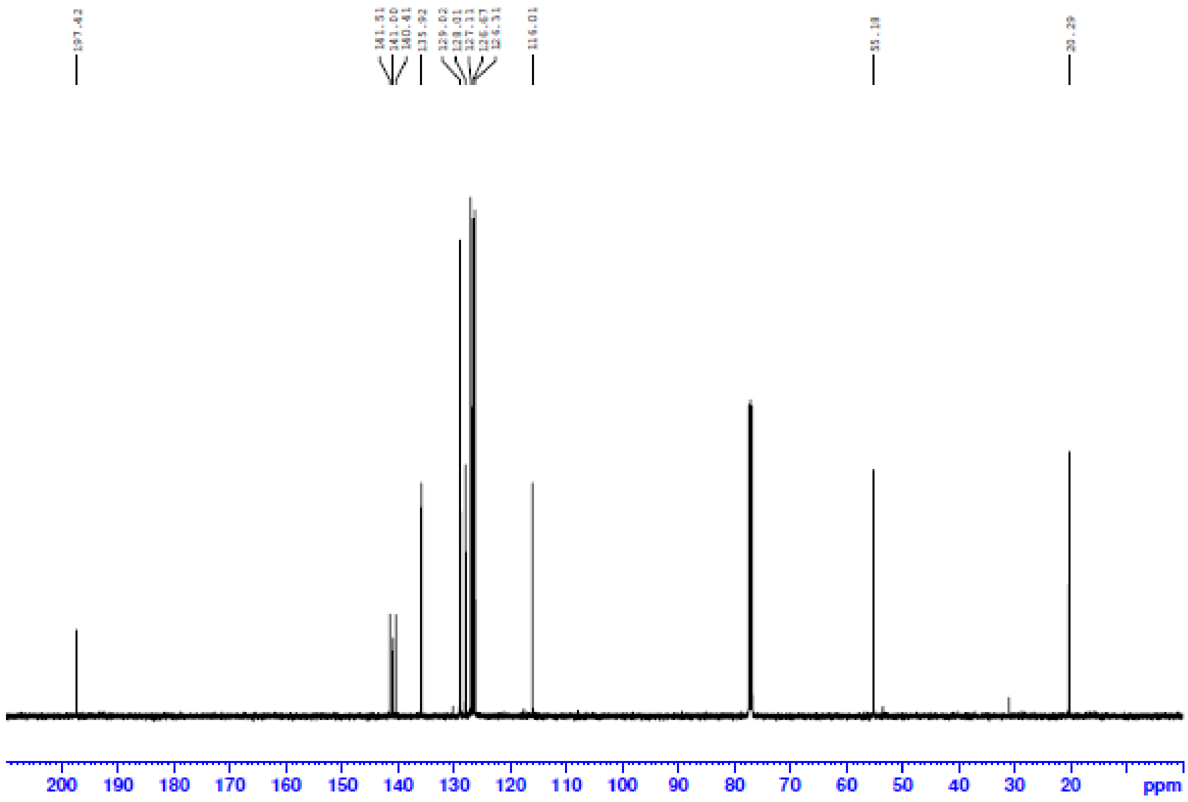

_N_-(9-Deoxyepicinchonidin-9-yl)-4-vinylbenzothioamide 5d

^{1}H NMR

Supporting Information

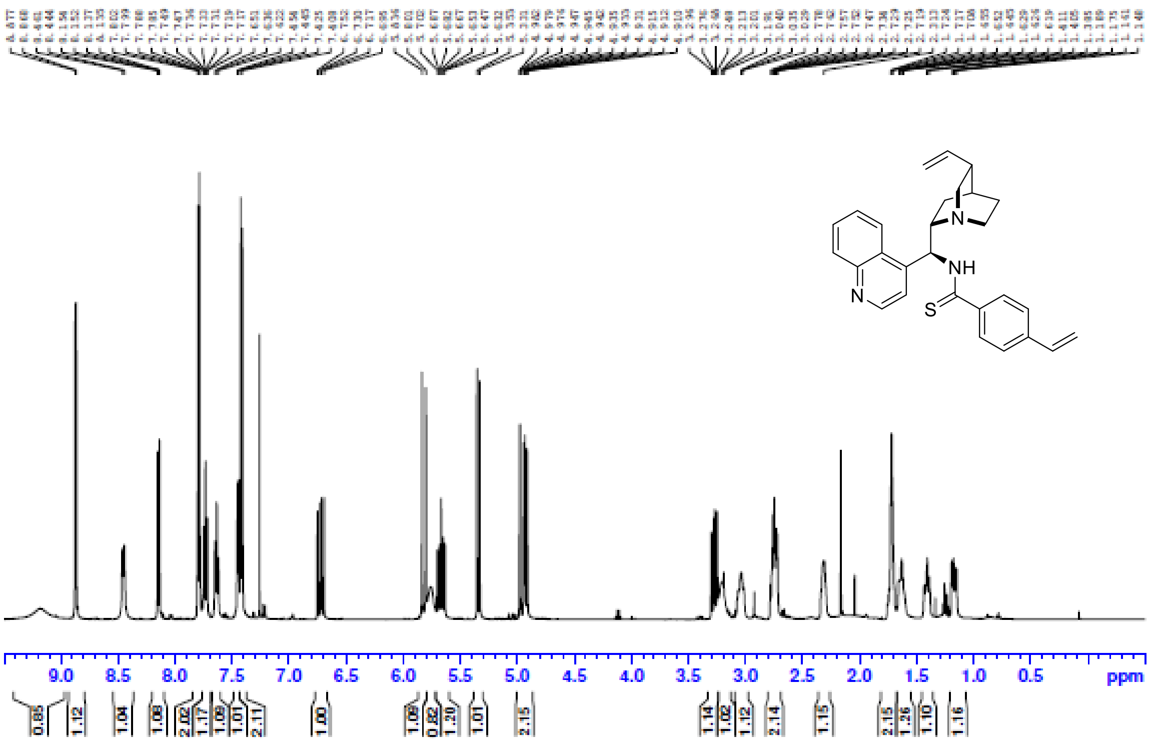

^{13}C NMR

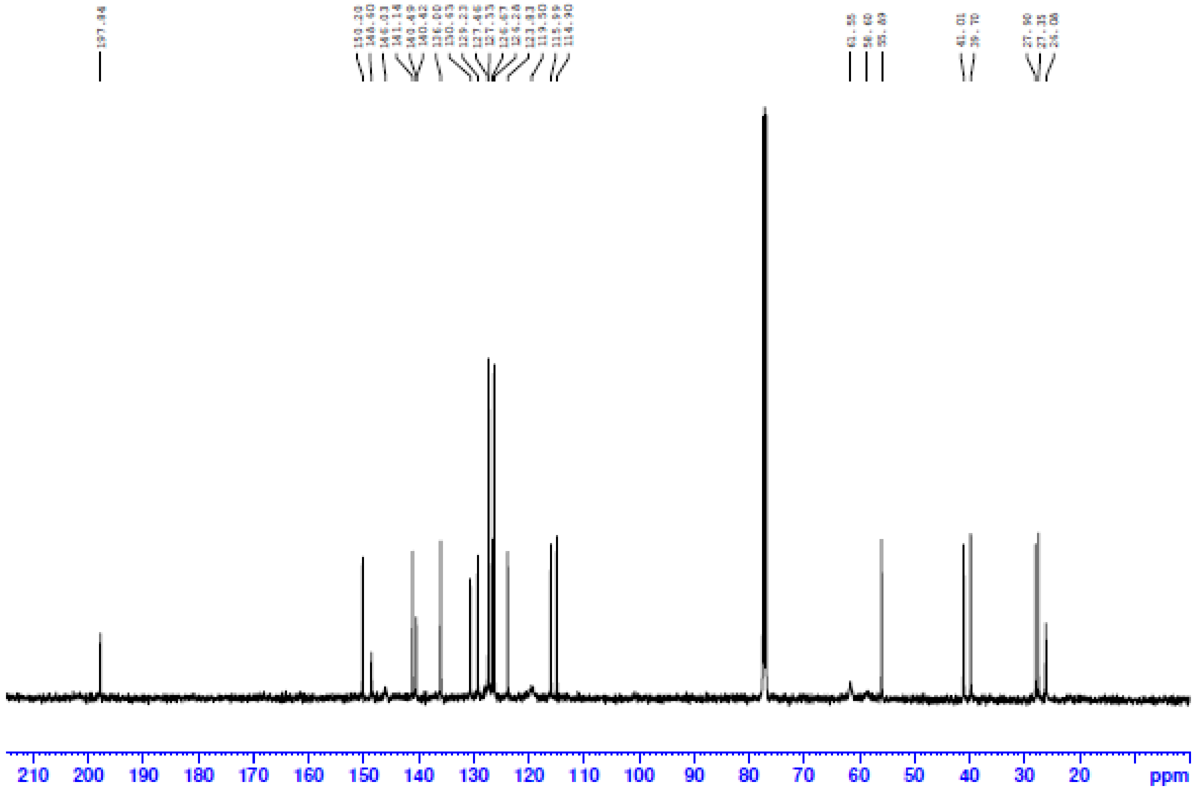

Supplement: Supplementary file 1 [file molecules-28-07333-s001.zip › molecules-2665352-supplementary.pdf]
